# Estrogen Receptor-α Suppresses Liver Carcinogenesis and Establishes Sex-Specific Gene Expression

**DOI:** 10.3390/cancers13102355

**Published:** 2021-05-13

**Authors:** Mara H. O’Brien, Henry C. Pitot, Sang-Hyuk Chung, Paul F. Lambert, Norman R. Drinkwater, Andrea Bilger

**Affiliations:** 1 Department of Craniofacial Sciences, University of Connecticut Health Center, 263 Farmington Avenue, Farmington, CT 06030, USA; obrien@uchc.edu; 2 McArdle Laboratory for Cancer Research, School of Medicine and Public Health, University of Wisconsin—Madison, 1111 Highland Ave, Madison, WI 53705, USA; pitot@oncology.wisc.edu (H.C.P.); lambert@oncology.wisc.edu (P.F.L.); norman.drinkwater@wisc.edu (N.R.D.); 3 Center for Nuclear Receptors and Cell Signaling, Department of Biology and Biochemistry, University of Houston, Houston, TX 77204, USA; schung2@Central.UH.EDU

**Keywords:** estrogen receptor, liver cancer, hepatocarcinogenesis, ovariectomy, sexual dimorphism, gene expression

## Abstract

**Simple Summary:**

The hormone estrogen is well known for its role in promoting breast and ovarian cancer. Estrogen has the opposite effect on the liver, which it protects from cancer. We show that this protection requires Estrogen Receptor α, but not Estrogen Receptor β, and correlates with a female pattern of liver gene expression. Female expression in the liver requires Estrogen Receptor α expressed in non-liver cells. Surgical removal of the ovaries, which protect females from liver cancer at least in part through their production of estrogen, does not affect the female-specific liver gene expression pattern. Estrogen may therefore influence liver carcinogenesis through multiple independent mechanisms. We identify six genes that are strong candidates for mediating *Esr1*′s protection against liver cancer.

**Abstract:**

Estrogen protects females from hepatocellular carcinoma (HCC). To determine whether this protection is mediated by classic estrogen receptors, we tested HCC susceptibility in estrogen receptor-deficient mice. In contrast to a previous study, we found that diethylnitrosamine induces hepatocarcinogenesis to a significantly greater extent when females lack *Esr1*, which encodes Estrogen Receptor-α. Relative to wild-type littermates, *Esr1* knockout females developed 9-fold more tumors. Deficiency of *Esr2*, which encodes Estrogen Receptor-β, did not affect liver carcinogenesis in females. Using microarrays and QPCR to examine estrogen receptor effects on hepatic gene expression patterns, we found that germline *Esr1* deficiency resulted in the masculinization of gene expression in the female liver. Six of the most dysregulated genes have previously been implicated in HCC. In contrast, *Esr1* deletion specifically in hepatocytes of *Esr1* conditional null female mice (in which Cre was expressed from the albumin promoter) resulted in the maintenance of female-specific liver gene expression. Wild-type adult females lacking ovarian estrogen due to ovariectomy, which is known to make females susceptible to HCC, also maintained female-specific expression in the liver of females. These studies indicate that *Esr1* mediates liver cancer risk, and its control of sex-specific liver gene expression involves cells other than hepatocytes.

## 1. Introduction

Liver cancer is the second leading cause of cancer death worldwide [1]. Men are twice as likely as women to develop liver cancer and to die from it [1,2]. The female hormone estrogen is well known as a cancer promoter in tissues such as the breast, endometrium, and ovary; but in the liver, estrogen is protective [3,4,5,6]. Epidemiological evidence suggests that liver cancer incidence is increased in postmenopausal females, that estrogen treatment suppresses liver cancer, and that the number of full-term pregnancies affects susceptibility to liver cancer [3,4,5]. In clinical trials, treating HCC patients with tamoxifen, which binds Estrogen Receptor α and blocks the effect of estrogen, resulted in a dose-dependent detrimental response with significantly fewer patients surviving for three months at higher doses [7]. The mechanism of estrogen’s protection is not well understood [3].

The mouse has been a useful model for understanding sex differences in the liver. As in humans, males are more susceptible to liver tumor development than females [8]. Ovariectomy increases liver tumor incidence in females, and castration reduces the incidence in males [9,10,11,12,13]. Thus, ovarian hormones protect against HCC, while androgens promote the growth of tumors. Studies indicate that estrogen acts at the promotion, rather than the initiation, stage of carcinogenesis [3,9,10,11,12,13]. There appears to be a critical period during which sex hormones are able to modify pathways regulating liver tumor susceptibility. The earlier the age at which ovariectomy is performed, the greater the increase in liver tumor development in females; and the earlier castration is performed, the greater the reduction in liver tumor development in males [13]. Chronic estrogen treatment of ovariectomized mice, after initiation with carcinogen, has been shown to decrease the levels of preneoplastic foci and liver tumors to the levels seen in intact females [14].

The Growth Hormone (GH) signaling pathway is responsible for most of the variation in hepatic gene expression seen between males and females. More continuous circulating GH levels result in a female gene expression pattern while pulsatile GH levels result in a male gene expression pattern [15]. Studies using mice with a deficiency in GH signaling have shown that these mice have greatly reduced liver tumor development following DEN-initiation, in both sexes, compared to wild-type (WT) animals [16]. In the absence of WT levels of GH, gonadectomy did not affect tumor outcome. These studies also showed that a male-specific gene expression pattern in the liver correlates with susceptibility to liver tumorigenesis.

It is unclear whether estrogen and its receptors affect sex-dependent tumor susceptibility and hepatic gene expression directly in the liver or indirectly, such as through the GH-regulated hypothalamic-pituitary-liver axis or through anti-inflammatory effects [17]. In addition, the roles of the estrogen receptor-coding genes *Esr1* (ERα) and *Esr2* (ERβ) in mediating estrogen’s effect are not well understood. A previous study analyzing the effect of *Esr1* on hepatocarcinogenesis in females found an increase in tumors in *Esr1* knockouts that was not significant; it concluded that *Esr1* does not affect hepatocarcinogenesis in females [18]. However, analyses of liver cancer gene expression in clinical samples and of gene function in cultured liver cancer cells suggest *Esr1* plays an important role in HCC. *Esr1* has been shown to suppress proliferation, migration, and invasion in liver cancer cells; its underexpression predicts a worse prognosis in HCC patients; and female liver cancers specifically upregulate a miRNA that reduces *Esr1* expression [19,20,21,22,23,24].

Our studies investigate the importance of *Esr1* and *Esr2* in HCC development in female mice, and show that *Esr1*, but not *Esr2*, is a key mediator of tumor outcome. We further examine the mechanism by which *Esr1* affects sex-dependent gene expression in the liver using a hepatocyte-specific knockout mouse model and show that *Esr1* regulates GH-dependent gene expression in the liver through an extrahepatic mechanism.

## 2. Results

### 2.1. Esr1 Protects against Liver Tumor Development

To determine whether *Esr1* influences HCC development, female *Esr1* KO, *Esr1* heterozygous, and WT littermates were treated with 0.1 µmol/g body weight DEN at 12 days of age and euthanized at 50 weeks. (This dose of DEN was chosen based on previous experiments analyzing the effect of sex hormones on hepatocarcinogenesis [16].) Analyses of liver tumor multiplicity and incidence (Table 1) show that *Esr1* has a strong protective effect. The multiplicity of liver tumors ≥ 1 mm in *Esr1* KO mice increased approximately 9-fold relative to WT littermates, and 5-fold relative to heterozygous littermates (*p <* 10^−9^ and *p* < 10^−7^, respectively). The multiplicity of liver tumors ≥ 5 mm was more than 10-fold higher in the *Esr1* KO mice relative to WT and heterozygous littermates (*p <* 10^−7^ and *p* < 10^−6^, respectively). There was no significant difference in tumor incidence for tumors ≥ 1 mm. However, *Esr1* KO mice had an increased incidence of tumors ≥ 5 mm compared to WT and heterozygous littermates (*p* < 0.0004 and *p* < 0.0005, respectively).

The stage of neoplastic disease was scored by our pathologist (H.C.P.). Overt tumors at least 1 mm in diameter from each group were selected at random for histological analysis; 45 were evaluated and 43 confirmed to be neoplastic. (The remaining two lesions were cystic or contained no distinct neoplasm and were removed from the analysis.) The proportion of adenoma and carcinoma subtypes was compared between *Esr1* genotypes using Fisher’s exact test (2-sided; Table 2). From the WT group we analyzed 10 tumors from 10 mice; from the *Esr1* heterozygous group we analyzed 14 samples from 10 mice; and from the *Esr1* KO group we analyzed 19 samples from 13 mice. *Esr1* KO females had significantly more carcinomas compared to heterozygous littermates (95% vs 57%; *p* < 0.026).

Similarly, *Esr1* KO females had more carcinomas than WT females (95% vs 70%). In contrast to the difference between heterozygotes and KO mice, the difference between WT and KO females was not significant (*p* > 0.10) likely due to the smaller number of WT lesions analyzed. Comparing all mice carrying at least one *Esr1* allele (*Esr1* +/− and *Esr1* WT) to *Esr1* KO mice yields a difference in carcinoma incidence of 62.4% vs 95%, which is significant (*p* < 0.027).

For female mice in the tumor study, the uterus was much smaller in *Esr1* KO mice compared to WT and heterozygous littermates, as shown previously (Supplementary Table S1; [25]). The female *Esr1* KO mice in the tumor study, unlike untreated *Esr1* KO females, had reduced body weight and approximately twice the liver weight and liver to body weight ratio.

Loss of *Esr2* did not alter the liver tumor phenotype (Table 3). Neither the multiplicity nor incidence of tumors ≥ 1 mm differed significantly. Similarly, neither the multiplicity nor incidence of the subset that were ≥ 5 mm differed significantly. *Esr2* mRNA is weakly expressed in the liver, and we found an 11-fold decrease in this transcript in *Esr2* KO females compared to female WT littermates. These results indicate that hepatic *Esr2* gene expression does not influence tumor susceptibility, and that estrogen-mediated protection against HCC depends on *Esr1*.

### 2.2. Esr1 Is Critical for Sexual Differentiation of Hepatic Gene Expression

**Figure 1:**
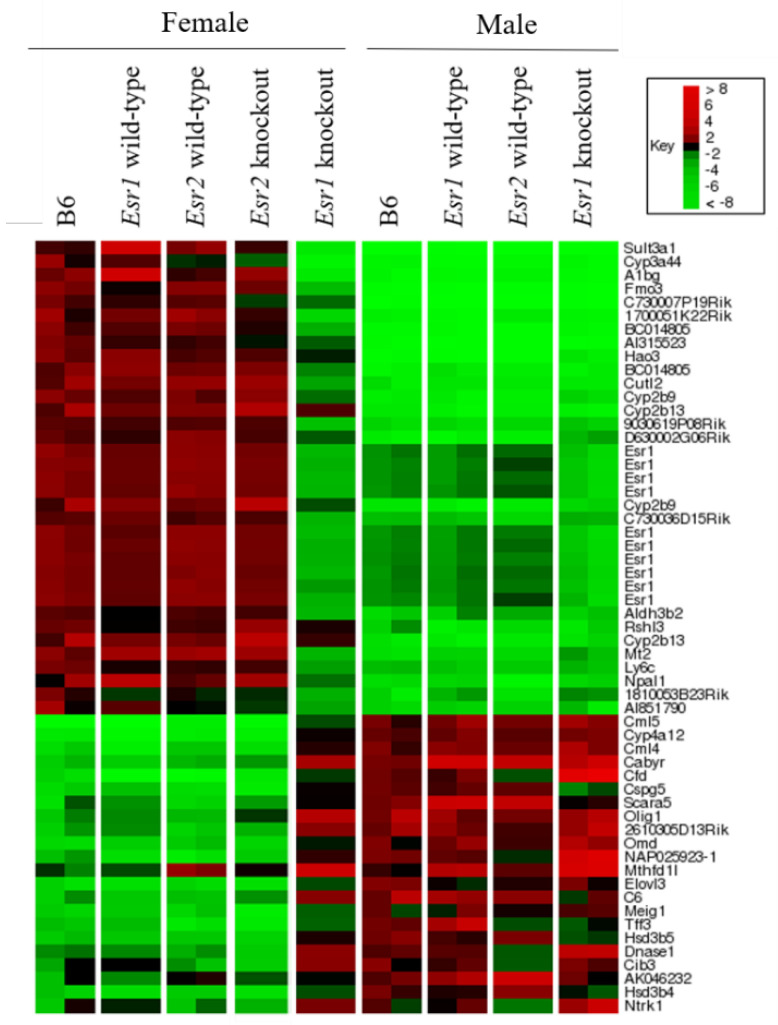
Estrogen Receptor-α knockout females, but not Estrogen Receptor-β knockout females, show male-specific liver gene expression. This heat map shows Estrogen Receptor-α (*Esr1*) regulated transcripts in female mice that differ at least 5-fold compared to wild-type (WT) female littermates. Each lane represents microarray results from a pool of livers from individual mice sacrificed at 9–10 weeks of age. Duplicate arrays are shown for each group. WT females are represented by the first three lanes. Lane 1: C57BL/6J (B6) females (*n* = 18). Lane 2: WT littermates of Estrogen Receptor-α knockout (*Esr1* KO) mice (*n* = 6). Lane 3: WT littermates of Estrogen Receptor-β knockout (*Esr2* KO) mice (*n* = 6). Lane 4: *Esr2* KO females (*n* = 8). Lane 5: *Esr1* KO females (*n* = 6). WT males are represented by lanes 6–8. Lane 6: B6 males (*n* = 15). Lane 7: WT littermates of *Esr1* KO mice (*n* = 6). Lane 8: WT littermates of *Esr2* KO mice (*n* = 6). Lane 9: *Esr1* KO males (*n* = 6).

To determine the effects of the *Esr1* and *2* deletions on gene expression, and to correlate these changes with susceptibility to HCC, we used microarrays to assess global gene expression in wild-type and knockout mice. We included the following groups, all on a B6 background: *Esr1* KO males and females and their wild-type littermates, *Esr2* KO females; control male mice, and control female mice. The ordered-list heat map in Figure 1 shows transcripts differing ≥ 5-fold between global *Esr1* KO females and WT females (*p* < 0.05).

Transcripts with increased and decreased expression in WT females, relative to *Esr1* KO females, are shown in red and green, respectively. Technical duplicate runs of the microarray are shown for each sample. Replicates had highly significant Spearman rank correlation coefficients of 0.74–0.94 (*p* = 4 × 10^−15^ to 3 × 10^−24^) per pair. B6 vs. *Esr1* WT, and B6 vs. *Esr2* WT comparisons for each sex also showed highly significant correlations, with rho = 0.85–0.90 (*p* = 3 × 10^−20^ to 1.6 × 10^−22^). In contrast, the correlation coefficient for B6 males and B6 females was −0.85.

The Agilent array used has 11 probes for the *Esr1* gene; 10 of these appear as two sets of green bars in *Esr1* KO mice, confirming their reduced expression. In the *Esr1* KO model, the production of full-length *Esr1* transcripts is abolished. However, smaller, alternatively spliced transcripts that produce smaller proteins are expressed. These proteins show no estrogen responsiveness, and the livers of *Esr1* KO females show no detectable specific ligand binding [26].

The heat map in Figure 1 shows that transcripts with higher expression in WT females, relative to WT males, tend to also have higher expression in WT females relative to *Esr1* KO females. Conversely, transcripts with higher expression in WT males relative to WT females also tend to have higher expression in *Esr1* KO females relative to WT females. There was no striking difference between *Esr1* WT males and *Esr1* KO males.

**Figure 2:**
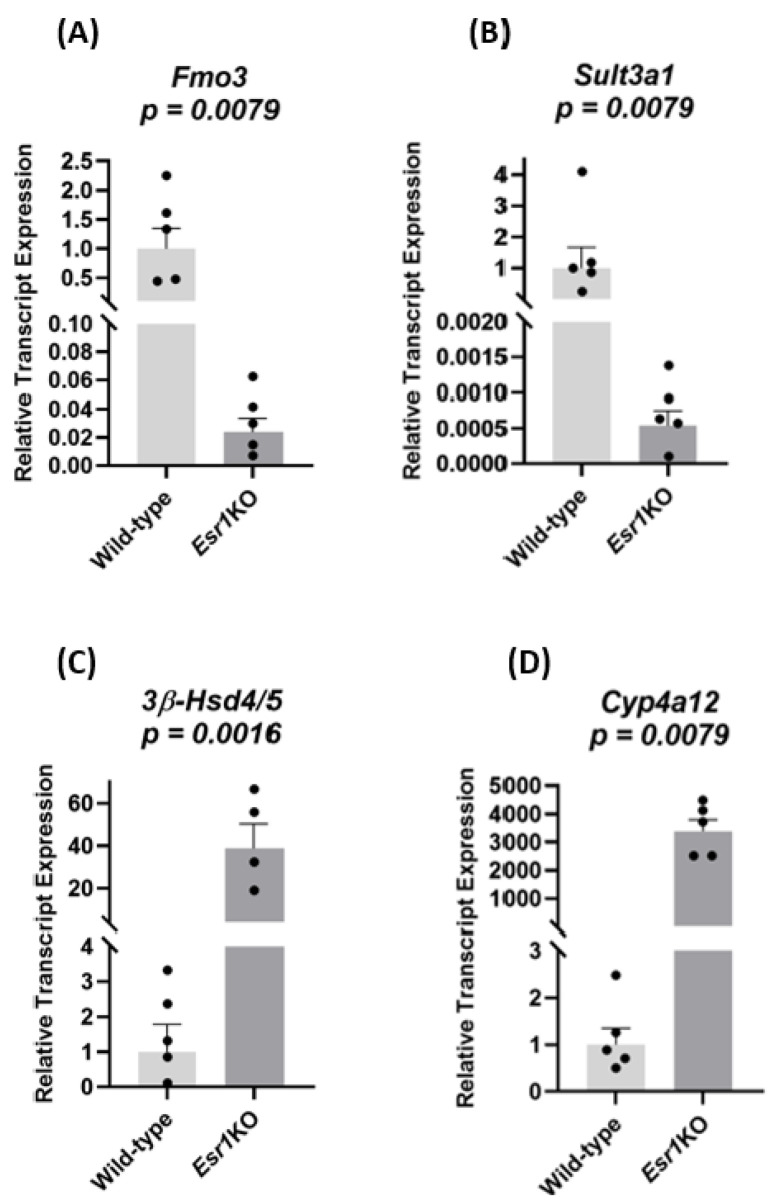
Pairwise analysis shows liver transcripts affected by *Esr1* knockout in females are largely sex-specific. Pairwise analysis showing log_2_ fold-change in transcript expression from microarray analysis comparing Estrogen Receptor-α (*Esr1*) dependent transcripts in females with sex-specific transcripts. All transcripts that changed at least two-fold in either comparison were included (*n* = 988). Each set of transcripts represents microarray results from a pool of livers from individual mice sacrificed at 9–10 weeks of age. Wild-type (WT) females are represented by a pool of C57BL/6J (B6) females (*n* = 18) and WT female littermates of the Estrogen Receptor-α KO mice (*n* = 6) and Estrogen Receptor-*β* KO mice (*n* = 6). Estrogen Receptor-α KO females are represented by a pool of intact individuals (*n* = 6) and females that underwent a sham operation (*n* = 6).

Expressed transcripts in *Esr1* KO females and B6 males have a high degree of overlap, relative to B6 females, with a rho for the Spearman correlation of 0.83 (*p* < 10^−148^; Figure 2). While most genes differed to approximately the same degree in *Esr1* KO females and B6 males, relative to B6 females, a few genes were found to have much more extreme differential expression between the sexes. Overall, these patterns of gene transcription are consistent with the known liver tumor susceptibility of B6 males relative to B6 females [8] and the observed susceptibility of *Esr1* KO females relative to WT females (Table 1).

The most differentially expressed *Esr1*-regulated transcripts and the corresponding sex-specific differential expression in B6 animals are shown in Supplementary Table S2. We found 45 unique transcripts to be differentially expressed ≥5-fold between *Esr1* KO females and WT females on the B6 background. Of these *Esr1*-regulated transcripts, 100% also differed at least 2-fold between the sexes in WT B6 mice (*p* < 0.05).

Among the 45 genes most differentially expressed between WT and *Esr1* KO mice, other than *Esr1*, 6 are strongly associated hepatocarcinogenesis: *A1bg*, *Fmo3*, *Cabyr*, *Cspg5*, *Mthfd1l*, *Tff3* (Table 4). *A1bg* and *Fmo3*, which are expressed more highly in WT females, are associated with protection. *A1bg* produces an antisense RNA that is under-expressed in HCC in patients with poor prognosis and reduces the malignancy of HCC cells when overexpressed [27]; *Fmo3* is one of 6 lipid-metabolism-related genes that together predict the prognosis of HCC patients [28]. *Cabyr*, *Cspg5*, *Mthfd1l* and *Tff3* are expressed more highly in *Esr1 KO* females and are associated with susceptibility. All are overexpressed in HCC. *Cabyr* also reduces cell proliferation when knocked down in liver cancer cells [29]; *Cspg5* is one of 6 genes that, together, predict HCC patient survival [30]; *Mthfd1l* predicts HCC patient survival [31]; *Tff3* also predicts HCC patient survival, and changing its expression in HCC cells affects the cells’ oncogenicity [32].

According to KEGG pathway enrichment analysis, transcripts induced by *Esr1*, with ≥3-fold greater expression in B6 females relative to *Esr1* KO females (Table 5), were involved in many pathways including calcium signaling and steroid hormone biosynthesis, and in the metabolism of drugs, xenobiotics, retinol, and arachidonic acid. Transcripts repressed by *Esr1*, with ≥3-fold greater expression in *Esr1* KO females relative to B6 females, were involved in a variety of pathways including mitogen-activated protein kinase (Mapk) signaling, extracellular-matrix-receptor interaction, endocytosis, the complement pathway, and the coagulation cascade.

**Figure 3:**
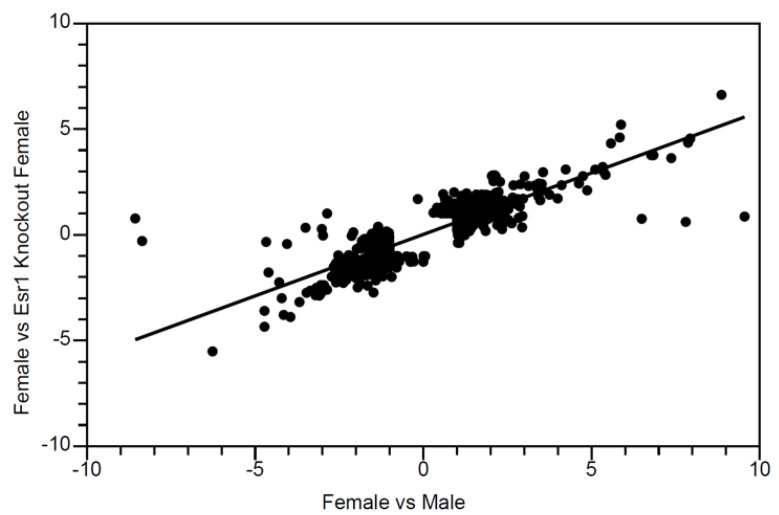
QPCR analysis of Estrogen Receptor-α-sensitive transcripts identified by microarray. RNA from the livers of 9–10 week-old mice was reverse transcribed and quantified by real-time PCR. WT females (*n* = 5) were littermates of the *Esr1* KO females. For *Fmo3*, *Sult3a1*, and *Cyp4a12* the *Esr1* KO female group was (*n* = 5) and for *3β-Hsd4/5* the *Esr1* KO female group was (*n* = 4). The average fold change and the standard error of the mean are shown. The ΔΔCT method was used to calculate the fold change and standard error of the mean. The Wilcoxon rank sum test (two-sided) was used to test for changes in gene expression where each transcript was analyzed individually.

To independently test the microarray results, we performed QPCR on reverse-transcribed, representative transcripts from *Esr1* KO and WT females (Figure 3). The female-specific transcripts flavin-containing *monooxygenase 3* (*Fmo3*) and *sulfotransferase 3a1* (*Sult3a1*) were expressed at higher levels in WT females than in *Esr1* KO females (Figure 3A, 1 ± 0.36 vs 0.024 ± 0.001; 3B, 1 ± 0.53 vs 0.00052 ± 0.00027; *p* < 0.008 for both). The male-specific transcripts *3β-hydroxysteroid* *dehydrogenase 4/5* (*3β-Hsd4/5*) and *cytochrome P450 4a12* (*Cyp4a12*) were expressed at lower levels in WT females than in *Esr1* KO females (Figure 3C, 1 ± 0.78 vs 41 ± 12, *p* < 0.02; D, 1 ± 0.30 vs 3400 ± 425, *p* < 0.008). These results are consistent with those of the microarray.

**Figure 4:**
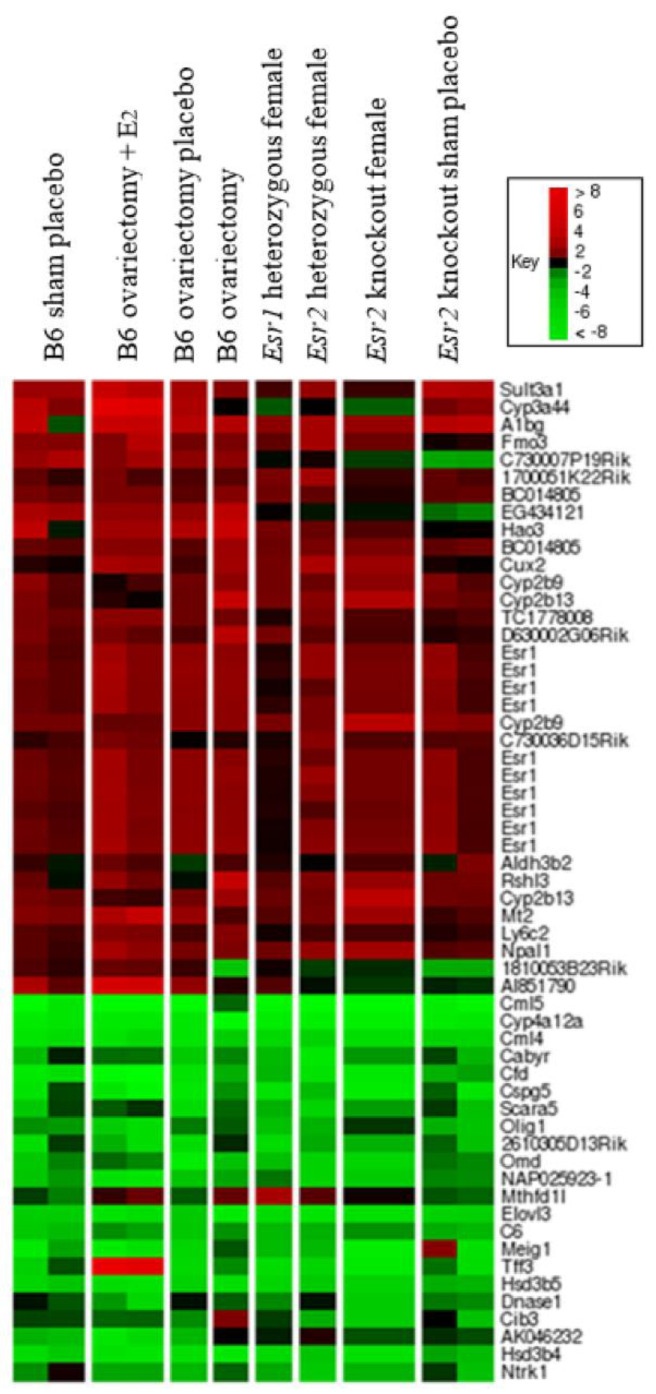
Estrogen Receptor-α-dependent gene expression is not altered in Estrogen Receptor heterozygotes, Estrogen Receptor-β knockout mice or in B6 females following alteration of ovarian hormones. Estrogen Receptor-α (*Esr1*) regulated transcripts in female mice showing at least 5-fold relative expression difference compared to wild-type (WT) female littermates are shown. Each lane represents microarray results from a pool of livers from individual mice sacrificed at 9–10 weeks of age. Duplicate arrays are shown for Lane 1: C57BL/6J (B6) sham operation placebo group (*n* = 7) and Lane 2: B6 ovariectomized animals with 17β-Estradiol add-back (*n* = 6) (Lane 2). A single array is shown for Lane 3: a pool of B6 females that underwent ovariectomy and implantation of a placebo pellet (*n* = 6), Lane 4: B6 animals with ovariectomy but no placebo implant (*n* = 5), Lane 5: *Esr*1 heterozygous females (*n* = 6), and Lane 6: *Esr*2 heterozygous females (*n* = 6). Duplicate arrays are shown for Lane 7: *Esr*2KO females (*n* = 8), and Lane 8: sham-operated *Esr*2KO females with placebo implants (*n* = 6).

Esr1-regulated transcripts were also examined in Esr1-heterozygous, Esr2-heterozygous, and Esr2 KO females. Gene expression in Esr1 heterozygous females was very similar to that in WT females, indicating that one copy of Esr1 is sufficient for normal sexual differentiation of gene expression in the liver (Figure 4). This gene expression pattern is consistent with the liver tumor resistance seen in Esr1 heterozygotes (Table 1 and Table 2). Gene expression in tumor-resistant Esr2 heterozygous females and Esr2 KO females (Table 3) also showed an overall normal WT female gene expression profile for Esr1-dependent genes (Figure 4).

Morphometric data and serum estrogen levels for animals used in the gene expression studies are shown in Supplementary Table S3. We found that female *Esr1* KO mice have a significantly lower uterus weight, higher body weight, and higher liver weight, as compared to heterozygous and WT littermates. The liver to body ratio was lower in *Esr1* KO and *Esr1* heterozygous females relative to WT females. There was no difference in the liver to body weight ratio between *Esr1* heterozygous and *Esr1* KO females. There were no significant differences in serum estrogen levels in *Esr1* KO females compared to WT females. *Esr1* heterozygous, *Esr2* heterozygous, and *Esr2* KO females were found to have a uterus weight and circulating estrogen levels that were similar to those seen in intact B6 animals. Global loss of *Esr1* in males did not alter body weight, liver weight, or the liver to body weight ratio.

### 2.3. Maintenance of Esr1-Dependent Gene Expression in Females Following Ovariectomy

To determine the role of ovarian hormones in establishing and maintaining sex-specific gene expression in adult livers, we assessed the effect of hormone depletion via ovariectomy and of hormone supplementation via 17β-estradiol pellets for two weeks on B6 mice. We compared the resulting gene expression pattern to the patterns seen in *Esr1* KO, *Esr2* KO and WT animals, using the ordered list heat map of transcripts that differed between *Esr1* KO and WT females (Figure 4). Surgical removal of the ovaries resulted in relatively few large gene expression changes. While transcripts of 45 *Esr1*-dependent genes were differentially expressed ≥ 5-fold in females, only 10 transcripts were found to be differentially expressed ≥ 5-fold following ovariectomy. All of these 10 transcripts showed higher expression in ovariectomized mice, indicating these genes are normally repressed by ovarian hormones.

Of the hepatic transcripts most dependent on *Esr1* signaling, very few were affected by hormone manipulation (Figure 4 and Supplementary Table S4). In B6 females, neither the ovariectomy group nor the ovariectomy plus 17β-estradiol add-back group had significantly altered *Esr1*-dependent signaling.

We further examined ovarian hormone-dependent gene transcription with ≥2-fold differences. Of the 63 transcripts that varied with ovariectomy, 48 were more abundant in ovariectomized females compared with intact animals (and are therefore normally repressed by ovarian hormones), while 15 were more abundant in intact animals (induced by ovarian hormones). Seventeen (27%) also differed between ovariectomized mice and mice that underwent ovariectomy plus 17β-estradiol add-back. Estrogen therefore regulates the expression of these transcripts in the intact mouse. None of the transcripts that changed at least 2-fold following ovariectomy showed *Esr1*-dependent or sex-dependent differential expression.

Similarly, 17β-estradiol treatment of ovariectomized animals changed only a very small fraction of the Esr1-dependent transcripts and sex-dependent transcripts (Supplementary Table S5). There were 87 genes that varied ≥2-fold between ovariectomized mice and mice that had ovariectomy plus 17β-estradiol add-back. Of these estrogen-dependent genes only 6 transcripts (7%) were *Esr1*-dependent, and 9 transcripts (10%) were sex-specific (Supplementary Table S4). Overall, transcripts that were associated with groups susceptible to liver tumors (WT males and *Esr1* KO females) were not those associated with the ovariectomy group (versus the 17β-estradiol treated ovariectomized group). This finding suggests that the adult estrogen-dependent protection seen in intact females may be due to a separate pathway than the congenital sex and *Esr1*-regulated pathways.

While ovarian estrogen is not required for most *Esr1*-dependent and sex-specific hepatic gene expression, it is required for maintenance of uterine weight. Ovariectomy caused a significant decrease in uterine weight (Supplementary Table S3). The uterine weight in ovariectomized B6 females was similar to that seen in susceptible *Esr1* KO females. Uterine weight was restored by providing ovariectomized B6 females with exogenous estrogen (*p* = 0.042 pairwise; *p* = 0.11 when adjusted for multiple comparisons [FDR]). Ovariectomy in B6 females reduced serum estrogen levels by half compared to intact females; however this difference was not significant (*p* = 0.77 pairwise; *p* = 0.97 corrected). Estrogen supplementation in ovariectomized mice in this experiment yielded circulating estrogen levels that were significantly elevated relative to physiological, non-pregnancy levels in intact mice.

Liver cancer-resistant *Esr1* heterozygous, *Esr2* heterozygous, and *Esr2* KO females were found to have a uterus weight similar to that seen in intact B6 animals and estrogen-treated ovariectomized B6 mice (Supplementary Table S3). *Esr1* heterozygous, *Esr2* heterozygous, and *Esr2* KO females also had serum estrogen levels that were not significantly different from those seen in intact B6 animals.

### 2.4. Regulation of Sex-Specific Hepatic Gene Expression by Esr1 Is Not Cell-Autonomous

**Figure 5:**
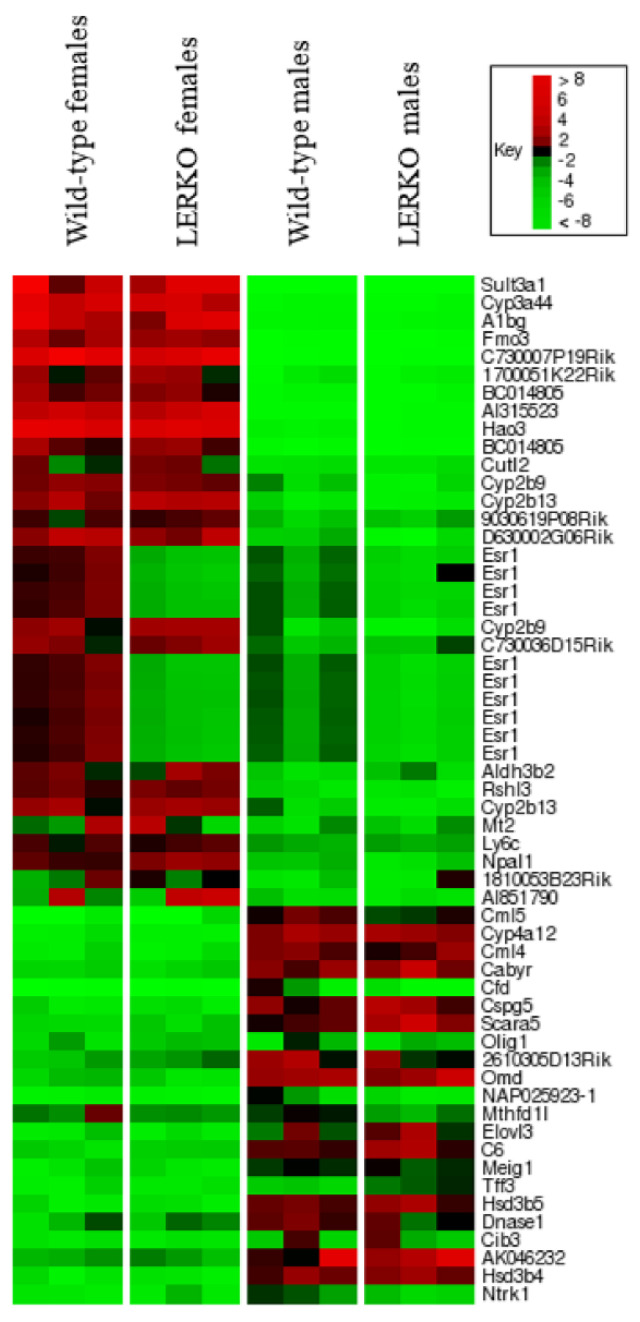
Hepatocyte-specific loss of Estrogen Receptor-α has little effect on sex-specific liver gene expression. This heat map shows gene expression associated with hepatocyte-specific loss of Estrogen Receptor-α (LERKO). Transcripts differing ≥ 5-fold in the global Estrogen Receptor-α knockout female group compared to WT females are shown. Each lane represents microarray results from individual mice sacrificed at 9–10 weeks of age. A single array was run for each animal. Lane 1: wild-type (WT) female littermates (*n* = 3). Lane 2: Female LERKO mice (*n* = 3). Lane 3: WT male littermates (*n* = 3). Lane 4: Male LERKO mice (*n* = 3).

To determine whether *Esr1*-mediated hepatic gene expression is a direct effect of *Esr1* in hepatocytes, we assessed hepatic gene expression in liver-specific *Esr1* KO (LERKO) animals on a mixed B6;FVB background. For these analyses we examined gene expression from three individual animals for each group: WT females, LERKO females, WT males, and LERKO males. The ordered list heat map in Figure 5 shows the effect of hepatocyte-specific *Esr1* deletion on the expression of the set of transcripts that differ more than 5-fold between global *Esr1* KO and WT females. (Of these hepatic transcripts regulated by global loss of *Esr1*, which were all sex-specific in B6 animals, 84% were also sex-specific in B6;FVB animals; Supplementary Table S2).

The expression of transcripts other than *Esr1* in LERKO females closely resembles the expression in WT females, and differs dramatically from that in males, indicating that the effect of *Esr1* on hepatic gene expression is indirect (Figure 5). The expression of male-specific transcripts other than *Esr1* was not affected, as expected based on the effect of global *Esr1* KO. Importantly, the expression of *Esr1* itself was dramatically reduced in LERKO females (6-fold) and males (3-fold), indicating that the Cre-driven excision was effective.

Although sex-dependent signaling remained similar to that of wild-type animals, many transcripts did depend on *Esr1* in hepatocytes. The changes in hepatic gene transcription seen in LERKO females, relative to WT females (B6;FVB background) were not as dramatic as in female global *Esr1* KO animals. Only 11 transcripts differed at least 5-fold, in contrast to the 45 transcripts that varied at least 5-fold between the global *Esr1* KO and WT animals (Supplementary Table S6). The transcript showing the greatest (27-fold) dependence on *Esr1*, *Ifi202b*, was previously shown to depend on *Esr1* expression in splenic cells [34]. This gene carries a promoter mutation in the B6 strain that reduces its expression to approximately 2% of the expression in other strains [34,35,36]; *Esr1* mutation reduces this expression further. None of the hepatic transcripts that varied in the LERKO animals was dependent on adult ovarian hormones.

*Esr1* was the only transcript found to vary in the LERKO females that also was sex-dependent and varied greater than 5-fold with global *Esr1* KO in females. Of 21 total transcripts that varied with LERKO in males greater than 5-fold, four of these (19%) were also sex-dependent. Two of these transcripts were also altered in the global *Esr1* KO females (Supplementary Table S6).

Seventy transcripts varied at least 2-fold in female LERKO mice, compared to WT female littermates. Among these transcripts there were differences in sex-specificity between the backgrounds. The expression of only one transcript (*Esr1*) showed 2-fold sex dependence in B6 mice, while 16 transcripts (23%) showed 2-fold sex dependence in B6;FVB mice (Supplementary Table S6). Thus, the vast majority of transcripts differentially expressed in LERKO mice are not sex specific. This indicates that the effect of *Esr1* on sex-specific hepatic gene expression is primarily through an indirect mechanism.

Also in contrast to what was seen in the global *Esr1* KO females, the uterus weight in LERKO animals was not significantly different from that of WT animals. The liver weight, and the liver to body ratio did not differ significantly between LERKO females and WT females. Loss of *Esr1* in males either globally or in hepatocytes specifically did not alter body weight, liver weight, or the liver to body weight ratio, compared to WT males (Supplementary Table S3).

## 3. Discussion

### 3.1. The Effects of Esr1, Esr2, Ovariectomy and Estradiol Supplementation on Gene Expression and Carcinogenesis

We found that *Esr1* (which encodes Estrogen Receptor α) dramatically protects female mice from liver tumor development. A single copy of *Esr1* is sufficient for protection. *Esr2* (Estrogen Receptor β) is normally weakly expressed or absent in the liver, and knockout of this gene did not affect hepatocarcinogenesis in females. The ability of *Esr1* to suppress liver carcinogenesis correlated with its ability to feminize gene expression in the liver. Indeed, most of the transcripts that differed between *Esr1* and WT females also differed between males and females. This effect on gene expression, like the effect on tumor resistance, was dominant: a single copy of *Esr1* was sufficient. Six of the sex-specific, *Esr1*-dependent transcripts are strongly implicated in hepatocarcinogenesis: *A1bg*, *Fmo3*, *Cabyr*, *Cspg5*, *Mthfd1l*, *Tff3*. Ovarian hormones had little effect on sex-dependent or *Esr1*-dependent gene expression.

Our results suggest that the effects of ovarian hormones on liver tumorigenesis in adult animals might be independent of any general effect of estrogen on *Esr1*-mediated liver gene expression. In these studies we show that neither ovariectomy, nor estrogen treatment of adult ovariectomized animals, greatly affects hepatic gene expression. Of the hepatic transcripts most dependent on *Esr1* signaling, very few were affected by circulating ovarian hormones in adult females. Exogenous estrogen strongly regulates *Tff3*, as does *Esr1*; however, ovariectomy reduces *Tff3* expression, while knocking out *Esr1* KO increases it. (An increase in *Tff3* is associated with HCC.)

It is possible that the *Esr1* pathway in the liver is affected in an estradiol-independent manner, either through ligand-independent mechanisms or through an alternative ligand, such as 27-hydroxycholesterol [37]. Alternatively, it is possible that ovarian hormones in adults also protect against carcinogenesis by feminizing liver gene expression, but that loss of feminine gene expression upon ovariectomy takes longer than the time we allowed between surgery and tissue collection (3 to 4 weeks). However, this time span was sufficient for ovariectomy to cause changes in uterine weight. We found that ovariectomy led to a reduction in circulating estrogen that was not statistically significant, likely due to the high variation of estrogen levels found in the intact B6 female group.

Ovarian hormones, which include estrogens, are known to protect against HCC in B6 mice [6]. Estrogens are derived from cholesterol, including 17β-estradiol, the most active form, and the weaker signaling molecules estrone and estriol. While the ovaries are the main source of estrogens in premenopausal females, estrogens can also be formed in adipose tissue and from adrenal androgens. Estradiol can be reversibly converted to estrone in various tissues, including the liver, by 17β-hydroxysteroid dehydrogenase. Estrogens can be inhibited by metabolism in the liver by sulfotransferases and UDP-glucuronosyltransferases.

In other studies, ovarian hormones have been shown to reduce inflammatory signaling associated with fatty liver [38]. Increased lipogenesis and reduced fatty acid oxidation occur following loss of ovarian hormones, resulting in hepatic lipid accumulation [39,40,41,42]. Fatty liver results when mice are fed a high-fat diet; it is associated with induction of proinflammatory cytokines [43,44]. Transcripts involved in lipid homeostasis have previously been shown to be elevated in ovariectomized rats when fed a diet containing 12.5% of calories provided by fat [45,46]. These changes in gene expression regulating inflammatory pathways could explain the susceptibility to HCC development seen in ovariectomized animals. In our studies, we did not observe changes in transcripts involved in lipid homeostasis in ovariectomized mice. This discrepancy may be due to differences in the fat content of the diet used in our study (9F 5020; 21.5% calories provided by fat), to the duration of the experiment, or to the species involved (rats vs. mice).

Genes significantly regulated by ovarian hormones that might play a role in hepatocarcinogenesis include *Edil3* (*EGF Like Repeats and Discoidin I-Like Domains 3*) and the *Myb* (*Myoblastosis*) oncogene. Overexpression of *Edil3*, which is upregulated in ovariectomized animals, is associated with HCC development by preventing anoikis [47]. The myeloblastosis oncogene (*Myb*) is upregulated in the livers of ovariectomized animals in breast cancer [48].

### 3.2. The Effect of Esr1, Ovariectomy and Estradiol Supplementation on Body Composition

We found, in addition to its role in liver carcinogenesis and liver gene expression, *Esr1* is required for normal female body mass. This observation confirms previous studies that showed that *Esr1* KO female animals have a significantly higher total body weight than WT animals [49]. However, *Esr1* KO animals in our tumor study had reduced body weight, which we attribute to HCC-associated pathology. The LERKO animals had normal body weights, indicating that *Esr1* regulates body weight at a site distinct from the liver. In contrast to its effect in females, *Esr1* does not regulate body weight in males.

It is unclear if the factors involved in the hypothalamic-pituitary-liver axis that govern hepatic gene expression and susceptibility of the liver to cancer development are similar to the pathways affecting body composition in females. Interpretation of hormonal action in the control of body composition is complicated, as the level of a single hormone can influence several pathways [50,51,52].

In B6 females we found that neither the ovariectomy group nor the ovariectomy plus 17β-estradiol add-back group of animals had body weights different than intact females. Ovariectomy has previously been shown in mice and rats to result in increased body weight, and this effect is blocked by treating with 17β-estradiol [46,53]. Possible explanations for this discrepancy include the fact that we did not use a high-fat diet, and there may have not been sufficient time following hormonal treatment in B6 females to see alterations in body weight.

The dose of estrogen used here, a 0.1 mg pellet, resulted in much higher serum estrogen levels than were seen in intact females. However, these levels were still below the serum estrogen levels (5000–10,000 pg/mL) associated with pregnancy [54]. The uterus weight was reduced in ovariectomized B6 animals, while the uterus weight and morphology in estrogen-treated ovariectomized females were similar to what was seen in females that underwent a sham operation (intact). In order to more closely mimic the intact female gene expression profile, it may be necessary to treat ovariectomized mice with progesterone as well. The hormonal fluctuations seen over the estrous cycle may also be important in regulating hepatic gene transcription and may play a role in protection against HCC.

The roles of sex hormones, *Esr1*, and *Esr2* in skeletal growth and homeostasis are reviewed in Callewaert et al. [55]. Estrogen has been shown to have a negative effect on bone accretion [56]; consistent with the increased body weight seen in the global *Esr1* KO females [49]. Prolactin is also important in regulating bone mineral density [57,58,59,60] and could be one of the factors altered.

### 3.3. Gluconeogenic and Lipogenic Effects in LERKO Mice

Both alcohol-associated steatosis [61] and non-alcohol associated fatty liver [62] are associated with increased incidence of HCC. Previous studies in an albumin-Cre-driven LERKO model have shown that LERKO male mice given a high-fat diet had reduced insulin sensitivity, and increased liver triglycerides and diacylglycerides, compared to WT littermates [63]. These differences between LERKO males and WT littermates were not seen in the absence of a high-fat diet. In contrast, in an alternative model where hepatic *Esr1* was deleted after development (using tail-vein injection of an adeno-associated construct carrying a liver-specific thyroxine-binding globulin-driven Cre), rather than early in development as in the albumin-Cre model, glucose and lipid metabolism were altered in LERKO male mice consuming a standard diet [64]. In our albumin-Cre LERKO model, fed a standard diet, we did not observe the changes in gluconeogenic genes (*Pck1* and *G6pc1*), and lipogenic genes (*Fasn* and *Acaca*) observed in the adeno-associated-virus-Cre model ‒ in mice of either sex. The observed differences in liver gene expression between mouse models may be due to the effects of hepatic *Esr1* during development. KEGG pathway enrichment analysis of differentially expressed LERKO genes in females in our study revealed no significant enrichment of gluconeogenic or lipogenic (or any other) pathways.

### 3.4. The Role of the GH-IGF Pathway in Sex-Specific Gene Expression and Carcinogenesis

The LERKO results indicate that *Esr1* signaling at an extrahepatic site affects sex-dependent liver gene expression and body growth. The extrahepatic effects of *Esr1* are likely to involve regulation of growth hormone (GH) levels, which in turn regulate gene expression in the liver [3,65]. GH is released from the anterior pituitary in a temporal pattern resulting in more continuous GH serum levels in females and more pulsatile GH in males. The length of time between GH pulses is believed to determine whether the gene expression profile in the liver is male [65]. Induction of GH-IGF (insulin-like growth factor) signaling is associated with increased cell division, increased body weight, increased bone and muscle mass, and an increase in metabolic activity and glucose utilization [66].

We have shown that *Esr1* is required for a female-specific liver gene expression pattern and protects female livers from carcinogen-induced tumor formation. We have previously shown that growth hormone shares these properties [16]. Our results confirm and extend those of others that suggest that Estrogen Receptor α and growth hormone lie in the same pathway leading to hepatocarcinogenesis [67].

GH regulation of gene expression in the liver is mainly mediated by the activity of Signal of Transducer and Activator of Transcription 5b (Stat5b) [67,68,69,70]. Stat5b is a transcription factor that is activated in response to the pulsatile GH pattern seen in males. Estrogen Receptor α is involved in the developmental imprinting mechanism that inhibits the nuclear localization of Stat5b in female mice, and *Esr1* KO females have previously been shown to express representative transcripts in a male GH-regulated pattern [67]. Analysis of *Stat5b* KO animals has shown that this factor can act as a hepatic oncogene or tumor suppressor gene depending on genetic background [70]. However, gene expression in the liver of each of these backgrounds was feminized, suggesting that general feminization of gene expression is not sufficient to confer resistance to hepatocarcinogenesis.

### 3.5. Alternative Mechanisms Behind Sex-Specific Liver Cancer Susceptibility: Inflammation, Metabolites

Studies have suggested that estrogen protects against liver cancer by blocking release of the growth factor Interleukin-6 (Il-6) by Kupffer cells following DEN exposure [71]. Il-6 is a key inducer of compensatory regeneration after carcinogen damage, when mutations can become fixed in the DNA and lead to tumor formation. In keeping with a possible role of estrogen in suppressing hepatic inflammation induced by Il-6, the 17β-estradiol mimic genistein, which binds Estrogen Receptor α, was found to reduce inflammation and increase apoptosis in DEN-treated mice [72]. These effects correlated with a reduced incidence of HCC. Estrogen Receptor α could exert such tumor-suppressive anti-inflammatory effects solely via stromal immune cells, indirectly affecting nascent neoplastic cells in the liver epithelium. Such extra-parenchymal effects have been observed in the cervix, where stromal Estrogen Receptor α is required to mediate the tumor-promoting effect of estradiol on the cervical epithelium [73,74]. Another possible mechanism of estrogen-mediated resistance to HCC may involve the hepatic metabolism of estrogen by Cytochrome P450 1a2, leading to protective metabolites that affect rates of proliferation and apoptosis in HCC [75].

### 3.6. A Previous Study Found No Significant Effect of Esr1

We were surprised to find such an influential role of *Esr1* in females, given that a previous study found the effect of *Esr1* on liver carcinogenesis was not significant [18]. These studies used the same *Esr1* KO model [25]. This model expresses no full-length *Esr1* transcript or peptide with detectable estradiol binding activity [26]. While smaller, alternatively spliced transcripts in this model yield peptides that could influence the tumor phenotype, such peptides should have been expressed in both studies.

The previous study of *Esr1* in the liver used a DEN dose of 20 mg/kg, approximately twice the dose used in our study [18]. The authors saw a 70% increase in tumor multiplicity in *Esr1* KO mice, but this increase was not significant. The high dose of carcinogen may have overwhelmed the ability of estrogen to suppress its effect—for example, by overwhelming estrogen’s ability to suppress DEN-induced local inflammation. Alternatively, the increased multiplicity of tumors induced by the higher dose of carcinogen might have interfered with accurate detection of individual tumors. The possibility that the effect of estrogen in these previous studies was systematically reduced or obscured is consistent with the authors’ observation of a 45% increase in tumorigenesis in ovariectomized mice compared to wild-type B6 mice that was also not significant. The effect of ovariectomy on hepatocarcinogenesis, including in the B6 strain, is well established [11,12,76]. Indeed, the authors saw a significant effect of ovariectomy in a hybrid B6-derived strain [18]. Thus it is difficult to interpret the meaning of their tumor studies in the context of protection by sex hormones.

### 3.7. Interplay between the Estrogen and Testosterone Pathways in Sex-Specific Liver Carcinogenesis

*Esr1* KO mice have higher testosterone levels than WT mice [76,77]. The susceptibility to HCC of *Esr1* KO females may be due in part to these elevated androgens. This possibility could be addressed by testing females lacking both estrogen receptor α and the androgen receptor for their susceptibility to liver cancer.

Studies have suggested, in mouse models and in liver cirrhosis patients, that the ratio of estrogen to testosterone may influence HCC susceptibility [3,78,79]. Bigsby and Caperell-Grant [18] have proposed that conversion of androgens to estrogen by steroid aromatase cytochrome P450 19a1, followed by activation of the *Esr1* pathway, may affect liver tumorigenesis. However, aromatase does not appear to be normally expressed in healthy liver tissues [80]. Human HCC tissues and liver cell lines show elevated aromatase, and it has been proposed that tumor promotion may occur through estrogen receptor regulation of growth pathways in these cells [81]. Thus, *Esr1* might protect against tumor development initially but then promote tumor growth in more advanced lesions.

### 3.8. Six Genes Dysregulated in Global Esr1 KO Females Are Implicated in Hepatocarcinogenesis

Six sex-specific, *Esr1*-dependent transcripts–*A1bg*, *Fmo3*, *Cabyr*, *Cspg5*, *Mthfd1l*, *Tff3*–are strongly implicated in hepatocarcinogenesis based on their expression, prognostic power, or oncogenic/tumor suppressive properties (Table 4). *A1bg* is a lncRNA recently shown to regulate the levels of PTEN and Smad7 [27]. *Fmo3* is a flavin-containing monooxygenase involved in lipid and drug metabolism [82]. *Cabyr* is a calcium binding protein and component of sperm flagella; in addition to HCC, it is expressed aberrantly in lung and brain cancers [83,84]. Its knockdown sensitizes cancer cells to TRAIL-induced apoptosis via Yap/p73-mediated regulation of Dr5 [84]. *Cspg5* affects neuronal differentiation [85]. *Mthfd1l* is involved in the synthesis of tetrahydrofolate, required for purine synthesis; its expression significantly correlates with the infiltration of immune cells including M2 (mainly anti-inflammatory) macrophages; and it appears to promote cell proliferation, migration, and invasion via the Akt/mTOR pathway [33,86]. *Tff3* facilitates migration and inhibits apoptosis, and its expression is increased in a variety of cancers [87]. This set of genes may be critical in mediating *Esr1*′s suppression of HCC, individually or in combination, and merits further study.

## 4. Materials and Methods

### 4.1. Mice

C57BL/6J (B6) mice were purchased from The Jackson Laboratory (Bar Harbor, ME, USA). *Esr1* and *Esr2* heterozygous mice on a B6 background were purchased from Taconic Laboratories (Hudson, NY, USA). The *Esr1* KO mice [25] and *Esr2* KO mice [88] were originally created by the Kenneth Korach laboratory at the National Institutes of Health. Breeding of male and female *Esr1* heterozygous mice generated the WT, *Esr1* heterozygous, and global *Esr1* KO mice used in these studies. Similar breeding of *Esr2* heterozygous mice was carried out to generate WT, *Esr2* heterozygous, and global *Esr2* KO mice.

To examine the effect of hepatocyte-specific effects we used mice with floxed *Esr1* inactivated by hepatocyte-specific Cre recombination driven by the albumin promoter, which causes *Esr1* to be inactivated specifically in hepatocytes during fetal development [89]. To generate these mice we obtained *Esr1*-flox mice, provided by Kenneth Korach (NIH), and backcrossed them to generation N8 on an FVB background [90]. We also obtained albumin-*Cre* mice provided by the Christopher Bradfield laboratory (University of Wisconsin). These mice were originally purchased from The Jackson Laboratory (stock #3574) and backcrossed to generation N21 or N22 on a B6 background. Albumin-*Cre* heterozygous mice (alb-*Cre*/+) were mated with B6 to give albumin-*Cre* heterozygotes. These albumin-*Cre* heterozygous (alb-*Cre*/+) mice were bred with *Esr1*-flox heterozygous (*Esr1*-flox/+) mice to generate *Esr1* heterozygous animals carrying the albumin-*Cre* transgene. These alb-*Cre*/ *Esr1*-flox/+ were then bred with other *Esr1*-flox heterozygous (*Esr1*-flox/+) mice to generate LERKO mice and their WT littermates, used in these studies.

Mice were housed in plastic cages on corncob bedding (Bed O’Cobs, Anderson Cob Division, Maumee, OH, USA). Animals were fed mouse diet 9F 5020 (Lab Diet, Madison, WI) and given acidified tap water ad libitum. Animals were inspected daily and all experimental protocols (under principal investigators N.R.D. and P.F.L.) were approved by the University of Wisconsin School of Medicine and Public Health Animal Care and Use Committee.

### 4.2. Genotyping

DNA was isolated from tail and toe clippings as previously described [91]. Amplification reactions were carried out using an MJ PTC-200 thermal cycler (Bio-Rad, Hercules, CA, USA). Products were visualized by electrophoresis through a 1.8% agarose gel and staining with ethidium bromide.

We used the following primers to genotype *Esr1*: ER2382 F: 5′-CGGTCTACGGCCAGTCGGGCATC-3′; Neo F: 5′-GCTGACCGCTTCCTCGTGCTTTAC-3′; Intron 2 Rev: 5′-CAGGCCTTACACAGCGGCCACCC-3′. PCR reaction conditions were as follows: 95 °C for 2 min, followed by 30 cycles of 95 °C for 30 s, 64 °C for 1 min, 72 °C for 1 min, and lastly an extension step at 72 °C for 7 min. DNA from WT mice leads to a 281 base pair product and DNA from *Esr1* KO mice leads to a 760 base pair product.

Genotyping of *Esr*2 has been described [92]. We used the following primers: Intron 2:5′-AGAATGTTGCACTGCCCCTGCTGC-3′; NeoBeta F: 5′-GCAGCCTCTGTTCCACAT ACACTTC-3′; Intron 3: 5′-GGAGTAGAAACAAGCAATCCAGACATC-3′. PCR conditions were as follows: 95 °C for 2 min, followed by 40 cycles of 94 °C for 45 s, 67 °C for 1 min, 72 °C for 1 min, and lastly an extension step at 72 °C for 7 min. DNA from WT mice leads to a 650 base pair product and DNA from *Esr2* KO mice leads to a 450 base pair product.

For genotyping *Esr1*-flox animals, we used primers N6delcKF: 5′- GACTCGCTACTGTGCCGTGTGC -3′; N6del3R: 5′-CTTCCCTGGCATTACCACTTCTCCT-3′. PCR conditions were as follows: 95 °C for 1 min, followed by 30 cycles of 94 °C for 30 s, 60 °C for 30 s, 72 °C for 30 s, and lastly an extension step at 72 °C for 5 min. DNA from WT mice leads to a 275 base pair product and DNA from *Esr1*-flox mice leads to a 475 base pair product.

Genotyping of the *Cre* transgene has been previously described [93]. We used the following primers: OL2642: 5′-TGCCTGCATTACCGGTCGATGC-3′; OL2643: 5′-CCATGAGTGAACGAACCTGGTCG-3′. PCR conditions were as follows: 95 °C for 5 min, followed by 25 cycles of 95 °C for 30 s, 60 °C for 30 s, 72 °C for 1 min, and lastly an extension step at 72 °C for 5 min. Presence of the Cre transgene leads to a 400 base pair product.

### 4.3. Induction of Liver Tumors and Analysis

To examine the effect of *Esr1* and *Esr2* on liver tumor development in female mice, we administered DEN (Sigma, St. Louis, MO, USA) at 12 ± 1 days of age with a single intraperitoneal injection (0.1 µmol/g body weight). Mice were sacrificed at 50 weeks with CO_2_ asphyxiation, livers were weighed, and tumors visible on the surface >1 mm in diameter were counted. Tumors were selected at random for histological analysis and were fixed in buffered formalin. Sections were stained with hematoxylin and eosin. Liver tumor incidence and multiplicity were compared using a two-sided Wilcoxon Rank Sum test, and the proportion of adenomas and carcinomas were compared with Fisher’s Exact test. Statistical tests were carried out with Mstat 6.62 software (Dr. Norman Drinkwater, University of Wisconsin, http://www.mcardle.wisc.edu/mstat/, accessed on 30 March 2020).

### 4.4. Hepatic Gene Expression Study

Mice were sacrificed at 9–10 weeks of age and weighed. The liver was dissected, rinsed in saline, weighed and placed in 10 mL of RNA Later (Qiagen, Valencia, CA, USA) in RNase-free conditions at 4 °C for at least 24 h. The liver was then transferred to an RNase-free microcentrifuge tube (Applied Biosystems, Foster City, CA, USA) and stored at −80 °C until RNA preparation was carried out using RNeasy Midi columns (Qiagen). RNA quality was assessed by electrophoresis on a 1% agarose gel, under RNase-free conditions, and also by spectrophotometric analysis using the ND-1000 NanoDrop instrument (Thermo-Fisher Scientific, Waltham, MA, USA). For females, the uterus was also weighed and blood was collected at sacrifice for serum preparation and measurement of estrogen levels using a radioimmunoassay. We did not measure serum estrogen levels in the LERKO females. Differences in morphometric traits and serum estrogen levels between groups were compared using the Wilcoxon Rank Sum Test (two-sided) with correction for multiple samples, using Mstat 6.62 software (30 March 2020).

For the WT female group we examined gene expression in RNA from pools of B6 individual females (*n* = 18), WT littermates of *Esr1* mice (*n* = 6), and WT littermates of *Esr2* mice (*n* = 6). For the *Esr1* KO female group we examined gene expression in RNA from pools of intact *Esr1* KO individuals (*n* = 6) and *Esr1* KO females that were exposed to a sham operation (*n* = 6). For the WT male group we examined gene expression in RNA from pools of B6 individual males (*n* = 15), WT littermates of *Esr1* mice (*n* = 6), and WT littermates of *Esr2* mice (*n* = 6). For characterization of gene expression in LERKO animals we used microarray analysis to examine mice individually (*n* = 3) from KO and WT littermates of each sex.

### 4.5. Ovariectomy and Estrogen Treatment

For studies involving surgeries, ovariectomy was performed or mice underwent a sham operation at 6 weeks of age and were allowed one week to recover. 17β-estradiol or placebo pellets were then implanted at 7 weeks of age. We used 0.1 mg 17β-estradiol pellets (Innovative Research of America, Sarasota, FL, USA) which are designed to release hormone over 90 days, resulting in a relatively constant hormone level. After exposure to the implant for 2 weeks, mice (9 to 10 weeks old) were euthanized by CO_2_ asphyxiation. Surgeries were performed under sterile conditions as described previously [94].

### 4.6. Serum Estrogen Levels

Blood was collected by cardiac puncture, transferred to microcentrifuge tubes, and allowed to clot at room temperature for at least 30 min. Serum was then prepared by centrifuging at 5000 rpm for 20 min and collecting the supernatant. The serum was stored at −20 °C and 17β-estradiol levels were measured using a radioimmunoassay kit (MP Biomedicals, Solon, OH, USA) according to the manufacturer’s instructions. The serum estrogen level is reported as the average value of replicates.

### 4.7. Quantitative Reverse Transcription-PCR

cDNA was synthesized from total RNA from livers of individual mice. 5 µg of RNA, resuspended in a total of 10 µl with RNase-free water, was used for reverse transcription using SuperScript II reverse transcriptase (Invitrogen, Carlsbad, CA, USA) according to manufacturer’s instructions. Oligo d(T)_15_ primers (Promega, Madison, WI, USA) and RNasin (Promega), were used in the reaction in a final volume of 20 uL. The NanoDrop was used to determine cDNA concentration, and cDNAs were diluted with water, to approximately 100 ηg/µL.

Primers were designed using Primer3 software [95] to generate amplicons in the 50–75 base pair range. Primers were ordered from Integrated DNA Technologies (Coralville, IA, USA). To test the efficiency of each primer set, we prepared standard curves ranging from approximately 410 ηg/ µL to 1.7 ηg/µL with cDNA pooled from several B6 female mice. Primer sets with reaction efficiencies between 1.8 and 2.2 were used in the analysis. Sequences of primers used in QPCR reactions are listed in Supplementary Table S7.

QPCR reactions were carried out in MicroAmp optical 96-well reaction plates with optical caps (Applied Biosystems). Each reaction was run in duplicate and consisted of 1 µL of the standard cDNAs, or the diluted sample cDNAs, along with 19 µL of the following mixture: 200 ηM of each primer, 3 mM MgCl2, 1X SYBR Green PCR buffer, 0.2 mM dNTPs with 0.4 mM dUTP, 0.2 U AmpErase UNG, 0.5 U AmpliTaq Gold DNA polymerase (SYBR Green PCR Core Reagents Kit, Applied Biosystems). The iCycler (Bio-Rad) was used to carry out QPCR with the following protocol; 50 °C for 2 min, 95 °C for 10 min, then 40 cycles of 95 °C for 15 s and 60 °C for 20 s. Melting curves, used to confirm reaction specificity, were started at 55 °C for 15 s, and the temperature was raised by 0.5 °C with each cycle, until 95 °C. As a control, each primer set was also tested in the absence of cDNA. Each primer set was compared to *β-actin* for normalization. The delta-delta Ct method was used to analyze relative transcript expression levels [96]. The mean fold-change and standard error of the mean was calculated, and the *p*-value was determined using the Wilcoxon Rank Sum test, using Mstat software.

### 4.8. Microarray, Heat Maps, Pathway Enrichment Analysis, and Functional Classification

For microarray analysis 1.3 µg of total RNA from each sample was used for fluorescent probe preparation, using the Agilent Low Input Linear Amplification and Labeling Kit (Agilent Technologies Inc., Santa Clara, CA, USA). Following linear amplification and dye labeling, the quality of samples was determined by measuring dye incorporation and cRNA concentration using the NanoDrop instrument. In the study examining the effect of deletion of *Esr1* specifically in the liver we examined gene expression in individual animals where probes were labeled with cyanine-3 (Cy-3) and the sex-reference control [pool of ½ B6 female (*n* = 18) and ½ B6 male (*n* = 15)] was labeled with cyanine-5 (Cy-5). In all other gene expression studies hepatic RNA was prepared from individual mice and RNA pools were made for each group, with approximately the same amount of RNA from each individual, using Cy-5 to label probes and Cy-3 to label the sex-reference control. Hybridizations were carried out according to the manufacturer instructions, using the Mouse Whole Genome 4 x 44 Oligo Microarray G4122F (Agilent Technologies Inc.), with 1.5 µg of labeled probe and 1.5 µg of labeled sex-reference control. The Agilent Microarray Scanner System G2565BA was used to measure hybridization signals and Agilent Feature Extraction software (version 9.1) was used for analysis. The data discussed in this publication have been deposited in NCBI’s Gene Expression Omnibus and are accessible through GEO Series accession number GSE164900 (https://www.ncbi.nlm.nih.gov/geo/query/acc.cgi?acc=GSE164900, accessed on 14 January 2021).

EDGE version 3 [97] was used to analyze differential expression data using a Linear Models for Microarray Data based statistical analysis with Benjamini-Hochberg correction. An adjusted *p*-value of less than 0.05 was considered significant. Transcripts with Cy-3 and Cy-5 processed signals of at least 100 were included in the analysis. Ordered list heat maps, with red used to show induction of gene expression and green used to show suppression of gene expression, were used to examine *Esr1*-regulated transcripts with ≥ 5-fold relative expression difference compared to WT females.

Enrichment of Kyoto Encyclopedia of Genes and Genomes (KEGG) pathways was conducted using WebGestalt software (http://www.webgestalt.org/, accessed on 6 April 2021) [98,99]. The list of gene IDs found to be regulated by *Esr1* with ≥ 3-fold relative expression difference compared to WT females was compared in the enrichment analysis, with the Agilent 4 x 44 whole mouse genome version 1 used as a reference. The hypergeometric test was used to evaluate significance of enrichment, using the Benjamini-Hochberg correction for multiple test adjustment. A *p*-value of less than 0.05 was considered significant.

## 5. Conclusions

We have shown that female mice are protected against liver tumor development by *Esr1* signaling. *Esr1* KO females have elevated body mass and a masculine liver gene expression pattern. Liver-specific *Esr1* KO females have a normal female body mass and a feminine liver gene expression pattern, indicating that *Esr1* regulates body mass and imprints the liver in female mice indirectly. It remains to be shown whether ovarian estrogen protects adult female mice against HCC development via *Esr1*, since we did not see similar changes in the gene expression pattern after ovariectomy as with a germline mutation of *Esr1*. Six genes highly dysregulated in *Esr1* KO mice have been implicated in HCC and may mediate the protection conferred by *Esr1*.

## Figures and Tables

**Table 1:** Effect of Estrogen Receptor-α on liver tumor multiplicity and incidence in females.

Mouse Strain	Tumor Multiplicity		Tumor Incidence		Number of Mice
	≥1 mm	≥5 mm	≥1 mm	≥5 mm	
Wild-type	4.1 ± 3.8	1.0 ± 1.6	28 (93%)	13 (45%)	30
*Esr1 +/−*	6.9 ± 8.6	1.5 ± 2.6	29 (94%)	14 (45%)	31
*Esr1 −/−*	36 ± 22 *	16 ± 12 *	23 (100%)	21 (91%) *	23

*Esr1*, Estrogen Receptor-α.Mean values ± standard deviation are shown. Within a size category, differences in tumor multiplicity between pairs of mouse strains were assessed using the Wilcoxon rank sum test, with FDR correction for multiple samples; differences in incidence were evaluated using Fisher’s exact test. * Significantly different from other two groups.

**Table 2:** Effect of Estrogen Receptor-α on tumor progression in females.

Mouse Strain	Adenomas	Carcinomas	Total	Percentage with Carcinomas
Wild-type	3	7	10	70% ^ab^
*Esr1 +/−*	6	8	14	57% ^a^
*Esr1 −/−*	1	18	19	95% ^b^

*Esr1*, Estrogen Receptor-α. Randomly selected tumors were fixed in buffered formalin, embedded, and sections were stained with hematoxylin and eosin. Fisher’s exact test (two-sided) was used to test for differences. Different letters indicate statistically significant differences between groups. *p* < 0.05 was considered significant.

**Table 3:** Effect of Estrogen Receptor-β on liver tumor multiplicity and incidence in females.

Mouse Strain	Tumor Multiplicity		Tumor Incidence		Number of Mice
	≥1 mm	≥5 mm	≥1 mm	≥5 mm	
Wild-type	8.7 ± 7.8	1.4 ± 3.0	26 (90%)	10 (34%)	29
*Esr2 +/−*	9.3 ± 6.8	2.0 ± 4.0	21 (100%)	11 (52%)	21
*Esr2 −/−*	11 ± 11	1.8 ± 3.4	24 (89%)	10 (37%)	27

*Esr2*, Estrogen Receptor-β. Mean values ± standard deviation are shown. Within a size category, differences in tumor multiplicity between pairs of mouse strains were assessed using the Wilcoxon rank sum test, with FDR correction for multiple samples, and differences in incidence were evaluated using Fisher’s exact test. *p* < 0.05 was considered significant.

**Table 4:** *Esr1*-regulated genes implicated in hepatocarcinogenesis.

Gene	*Esr1* KO Expression	HCC Expression	Evidence for Role in HCC	Reference
*A1bg*	Down	Down	Reduced expression associated with poor prognosis; expression reduces oncogenicity in vitro	[27]
*Fmo3*	Down	Down	Part of 6-gene lipid metabolism-related prognostic signature	[28]
*Cabyr*	Up	Up	Knockdown reduces proliferation	[29]
*Cspg5*	Up	Up	Part of 6-gene prognostic signature	[30]
*Mthfd1l*	Up	Up	Predicts patient survival; correlates with immune infiltration	[31,33]
*Tff3*	Up	Up	Predicts patient survival; increases oncogenicity in vitro and in vivo	[32]

**Table 5:** KEGG pathway enrichment: Transcripts regulated by Estrogen Receptor-α.

KEGG Pathway Enriched	WT Females vs. *Esr1 −/−* Females				
Transcripts Higher in Wild-Type Females	Symbol	Fold Change	Fold-Change Adjusted *p*-Value	Ratio of Enrichment	Enrichment Adjusted *p*-Value
Drug metabolism–cytochrome P450	*Cyp2b10*	4.3	*p* = 5.39 × 10^−8^	104	1.33 × 10^−9^
	*Cyp3a44*	37	*p* = 1.07 × 10^−13^		
	*Cyp2b13*	8.0	*p* = 1.59 × 10^−7^		
	*Fmo3*	24	*p* = 1.38^−11^		
	*Cyp2b9*	8.5	*p* = 1.24 × 10^−13^		
Metabolism of ×enobiotics by	*Cyp2b10*	4.3	*p* = 5.39 × 10^−8^	93	1.01 × 10^−7^
cytochrome P450	*Cyp3a44*	37	*p* = 1.07 × 10^−13^		
	*Cyp2b13*	8.0	*p* = 1.59 × 10^−7^		
	*Cyp2b9*	8.5	*p* = 1.24 × 10^−13^		
Retinol metabolism	*Cyp2b10*	4.3	*p* = 5.39 × 10^−8^	93	1.01 × 10^−7^
	*Cyp3a44*	37	*p* = 1.07 × 10^−13^		
	*Cyp2b13*	8.0	*p* = 1.59 × 10^−7^		
	*Cyp2b9*	8.5	*p* = 1.24 × 10^−13^		
Metabolic pathways	*Cyp2b10*	4.3	*p* = 5.39 × 10^−8^	12	2.04 × 10^−7^
	*Sqle*	3.2	*p* = 3.33 × 10^−3^		
	*Cyp3a44*	37	*p* = 1.07 × 10^−13^		
	*Cyp2b13*	8.0	*p* = 1.59 × 10^−7^		
	*Hao3*	12	*p* = 3.11 × 10^−8^		
	*Cyp2b9*	8.5	*p* = 1.24 × 10^−13^		
	*Nt5e*	5.0	*p* = 1.76 × 10^−10^		
	*Akr1b7*	3.4	*p* = 3.98 × 10^−6^		
Arachidonic acid metabolism	*Cyp2b10*	4.3	*p* = 5.39 × 10^−8^	60	1.75 × 10^−5^
	*Cyp2b13*	8.0	*p* = 1.59 × 10^−7^		
	*Cyp2b9*	8.5	*p* = 1.24 × 10^−13^		
Steroid hormone biosynthesis	*Akr1c18*	3.1	*p* = 1.62 × 10^−3^	66	0.0004
	*Cyp3a44*	37	*p* = 1.07 × 10^−13^		
Calcium signaling pathway	*Avpr1a*	3.9	*p* = 4.49 × 10^−9^	20	0.0044
	*Atp2b2*	4.1	*p* = 1.28 × 10^−5^		
Transcripts higher in *Esr1*KO females					
Prion diseases	*Hspa1a*	3.6	*p* = 1.68 × 10^−3^	70	0.0004
	*C6*	6.3	*p* = 1.45 × 10^−12^		
Complement and coagulation	*Cfd*	12	*p* = 4.57 × 10^−3^	32	0.0018
cascades	*C6*	6.3	*p* = 1.45 × 10^−12^		
ECM-receptor interaction	*Lama3*	4.3	*p* = 6.03 × 10^−8^	29	0.0023
	*Col5a3*	3.1	*p* = 4.16 × 10^−4^		
Metabolic pathways	*Mthfd1l*	6.7	*p* = 2.72 × 10^−8^	5.2	0.0027
	*Hsd3b5*	5.9	*p* = 9.16 × 10^−12^		
	*9130409I23Rik*	3.8	*p* = 8.23 × 10^−8^		
	*Cyp4a12*	20	*p* = 6.31 × 10^−18^		
	*Alas2*	4.8	*p* = 2.92 × 10^−16^		
Amoebiasis	*Lama3*	4.3	*p* = 6.03 × 10^−8^	21	0.0041
	*Col5a3*	3.1	*p* = 4.16 × 10^−4^		
To×oplasmosis	*Lama3*	4.3	*p* = 6.03 × 10^−8^	19	0.0048
	*Hspa1a*	3.6	*p* = 1.68 × 10^−3^		
Focal adhesion	*Lama3*	4.3	*p* = 6.03 × 10^−8^	12	0.0116
	*Col5a3*	3.1	*p* = 4.16 × 10^−4^		
Endocytosis	*Ntrk1*	5.0	*p* = 7.94 × 10^−4^	11	0.0139
	*Hspa1a*	3.6	*p* = 1.68 × 10^−3^		
MAPK signaling pathway	*Ntrk1*	5.0	*p* = 7.94 × 10^−4^	9	0.0201
	*Hspa1a*	3.6	*p* = 1.68 × 10^−3^		
Neuroactive ligand-receptor	*Mtnr1a*	3.4	*p* = 2.48 × 10^−3^	9	0.0214
interaction	*Grm8*	3.4	*p* = 7.08 × 10^−3^		

KEGG, Kyoto Encyclopedia of Genes and Genomes; *Esr1*KO, estrogen receptor-α knockout. Fold change represents microarray results from hepatic RNA of mice sacrificed at 9–10 weeks of age. The adjusted P-value for each transcript is listed. The female wild-type (WT) group is composed of C57BL/6J (B6) females (*n* = 18) and the wild-type female littermates of the estrogen receptor-α (*n* = 6) and estrogen receptor-β mice (*n* = 6). *Esr1* KO females are represented by intact individuals (*n* = 6) and females that underwent a sham operation (*n* = 6).

## Data Availability

The data discussed in this publication have been deposited in NCBI’s Gene Expression Omnibus and are accessible through GEO Series accession number GSE164900, accessed on 14 January 2021.

## Supplementary Materials

The following are available online at https://www.mdpi.com/article/10.3390/cancers13102355/s1, Table S1: Morphometric analysis of mice used in tumor study, Table S2: Transcripts with at least 5-fold differential expression between wild-type females compared to global estrogen receptor-α knockout females and corresponding sex-specific expression, Table S3: Morphometric analysis of mice used in gene expression study, Table S4: Transcripts expressed differentially in C57BL/6J females between the ovariectomized group vs the ovariectomy plus 17β-estradiol add-back group and corresponding sex-specific and estrogen receptor-α dependent expression, Table S5: Ovarian hormone-dependent transcripts in C57BL/6J mice which are also dependent on 17β-estradiol, Table S6: Transcripts with at least 2-fold differential expression between LERKO females and wild-type females and corresponding sex-specific expression, Table S7: Quantitative real-time PCR primers used to measure hepatic mRNA levels in global estrogen receptor-α knockout females and wild-type littermates.
