# Supplementary material for: Estrogen Receptor-α Suppresses Liver Carcinogenesis and Establishes Sex-Specific Gene Expression
Source: Cancers (Basel). 2021 May 13;13(10):2355. doi: 10.3390/cancers13102355 (PMC8153146; doi:10.3390/cancers13102355)
Supplement: Supplementary file 1 [file cancers-13-02355-s001.zip › cancers-1196403-supplementary.pdf]

cancers

MDPI

Supplementary Materials: Estrogen Receptor-α suppresses liver carcinogenesis and establishes sex-specific gene expression

Table S1. Morphometric analysis of mice used in tumor study.

	Number of mice	Body weight (g)	Liver weight (g)	Liver/body weight	Uterus weight (mg)
Wild-type (*Esr1* littermates)	30	36.4 ± 5.5 [a]	1.74 ± 0.57 [a]	0.050 ± 0.024 [a]	125 ± 38 [a]
Esr1 +/−	31	35.5 ± 6.0 [ab]	1.86 ± 0.69 [a]	0.053 ± 0.021 [a]	129 ± 70 [a]
Esr1 −/−	23	32.8 ± 5.9 [b]	4.26 ± 1.94 [b]	0.132 ± 0.059 [b]	40 ± 27 [b]
Wild-type (*Esr2* littermates)	29	37.1 ± 6.2 [a]	2.06 ± 1.39 [a]	0.058 ± 0.050 [a]	173 ± 70 [a]
Esr2 +/−	21	40.2 ± 5.3 [ab]	1.79 ± 0.53 [a]	0.046 ± 0.020 [a]	136 ± 51[a]
Esr2 −/−	27	43.3 ± 7.4 [b]	2.21 ± 1.01 [a]	0.052 ± 0.023 [a]	142 ± 62 [a]

Abbreviations: *Esr1*, estrogen receptor-α; *Esr2*, estrogen receptor-β. Body weight, liver weight, liver/body ratio, and uterus weight are shown as the mean ± standard deviation. Statistical analysis was carried out for the *Esr1* and *Esr2* groups separately. Pairwise comparison using Wilcoxon rank sum test with correction for multiple samples (FDR) was used to test for differences. $p < 0.05$ was considered significant. Different letters indicate statistically significant differences between groups.

Table S2. Transcripts with at least 5-fold differential expression between wild-type females compared to global estrogen receptor-α knockout females and corresponding sex-specific expression.

	(B6 Background) WT Females vs. *Esr1*KO Females		(B6 Background) WT Females > WT Males		(B6;FVB Background) WT Females > WT males	
Higher in WT females	Fold Change	Adjusted *p* - value	Fold Change	Adjusted *p* - value	Fold Change	Adjusted *p* - value
1) *Sult3a1*	98	$p = 1.14\times10^{-14}$	466	$p = 2.68\times10^{-24}$	1129	$p = 2.05\times10^{-13}$
2) *Cyp3a44*	37	$p = 1.07\times10^{-13}$	59	$p = 1.03\times10^{-19}$	213	$p = 9.31\times10^{-13}$
3) *A1bg*	24	$p = 1.33\times10^{-9}$	57	$p = 7.82\times10^{-17}$	145	$p = 1.98\times10^{-9}$
4) *Fmo3*	23	$p = 1.38\times10^{-11}$	245	$p = 8.47\times10^{-26}$	265	$p = 5.79\times10^{-13}$
5) C730007P19Rik	20	$p = 9.02\times10^{-7}$	234	$p = 4.37\times10^{-19}$	1163	$p = 1.01$
6) 1700051K22Rik	20	$p = 1.90\times10^{-11}$	48	$p = 1.24\times10^{-19}$	26	$p = 1.33\times10^{-6}$
7) BC014805	14	$p = 3.07\times10^{-10}$	110	$p = 1.50\times10^{-24}$	54	$p = 2.98\times10^{-9}$
8) AI315523	14	$p = 4.08\times10^{-19}$	115	$p = 3.63\times10^{-23}$	192	$p = 2.51\times10^{-12}$
9) *Hao3*	12	$p = 3.11\times10^{-8}$	166	$p = 4.90\times10^{-24}$	157	$p = 1.68\times10^{-11}$
10) *Cutl2*	8.5	$p = 1.96\times10^{-11}$	19	$p = 1.00\times10^{-20}$	7.3	$p = 2.93\times10^{-5}$
11) *Cyp2b9*	8.5	$p = 1.24\times10^{-13}$	35	$p = 2.16\times10^{-27}$	8.3	$p = 1.95\times10^{-6}$
12) *Cyp2b13*	8.0	$p = 1.59\times10^{-7}$	41	$p = 3.56\times10^{-20}$	23	$p = 1.86\times10^{-6}$
13) 9030619P08Rik	7.8	$p = 6.19\times10^{-19}$	12	$p = 1.38\times10^{-27}$	5.1	$p = 1.38\times10^{-7}$
14) D630002G06Rik	7.1	$p = 1.54\times10^{-7}$	43	$p = 1.67\times10^{-21}$	14	$p = 1.16\times10^{-5}$
15) *Esr1*	7.0	$p = 1.53\times10^{-16}$	4.5	$p = 4.71\times10^{-15}$	3.1	$p = 8.63\times10^{-4}$
16) C730036D15Rik	6.8	$p = 4.71\times10^{-14}$	8.0	$p = 1.96\times10^{-19}$	4.8	$p = 6.09\times10^{-5}$
17) *Aldh3b2*	5.7	$p = 4.96\times10^{-8}$	4.9	$p = 1.15\times10^{-9}$	8.7	$p = 2.00\times10^{-5}$

18) *Rshl3*	5.4	$p = 4.13 \times 10^{-7}$	11	$p = 3.77 \times 10^{-15}$	11	$p = 2.59 \times 10^{-6}$
19) *Mt2*	5.3	$p = 0.0013$	11	$p = 2.05 \times 10^{-9}$	N/A	$p > 0.05$
20) *Ly6c*	5.2	$p = 1.35 \times 10^{-15}$	7.4	$p = 3.00 \times 10^{-23}$	3.6	$p = 5.33 \times 10^{-5}$
21) *Npal1*	5.1	$p = 2.60 \times 10^{-7}$	17	$p = 1.98 \times 10^{-19}$	5.5	$p = 1.05 \times 10^{-3}$
22) 1810053B23Rik	5.1	$p = 0.0068$	6.4	$p = 2.04 \times 10^{-5}$	N/A	$p > 0.05$
23) AI851790	5.0	$p = 0.00015$	10	$p = 6.58 \times 10^{-11}$	4.9	$p = 0.044$
	**(B6 background) WT females vs. *Esr1*KO females**		**(B6 background) WT males > WT females**		**(B6;FVB background) WT males > WT females**	
**Higher in *Esr1*KO females**	**Fold Change**	**Adjusted *p*-value**	**Fold Change**	**Adjusted *p*-value**	**Fold Change**	**Adjusted *p*-value**
1) *Cml5*	46	$p = 3.07 \times 10^{-10}$	77	$p = 9.96 \times 10^{-16}$	71	$p = 4.90 \times 10^{-5}$
2) *Cyp4a12*	20	$p = 6.31 \times 10^{-18}$	26	$p = 4.62 \times 10^{-24}$	29	$p = 1.05 \times 10^{-6}$
3) *Cml4*	15	$p = 7.37 \times 10^{-19}$	15	$p = 7.69 \times 10^{-24}$	18	$p = 5.08 \times 10^{-8}$
4) *Cabyr*	14	$p = 1.17 \times 10^{-13}$	18	$p = 4.34 \times 10^{-19}$	12	$p = 1.79 \times 10^{-5}$
5) *Cfd*	12	$p = 0.0046$	26	$p = 5.59 \times 10^{-7}$	30	$p = 0.046$
6) *Cspg5*	9.1	$p = 1.83 \times 10^{-6}$	13	$p = 4.36 \times 10^{-11}$	15	$p = 1.95 \times 10^{-3}$
7) *Scara5*	7.7	$p = 3.74 \times 10^{-6}$	19	$p = 1.38 \times 10^{-13}$	9.1	$p = 0.011$
8) *Olig1*	7.1	$p = 8.21 \times 10^{-10}$	8.5	$p = 2.41 \times 10^{-14}$	N/A	$p > 0.05$
9) 2610305D13Rik	7.1	$p = 7.23 \times 10^{-7}$	9.1	$p = 4.28 \times 10^{-11}$	7.7	$p = 2.31 \times 10^{-3}$
10) *Omd*	7.1	$p = 9.92 \times 10^{-10}$	8.3	$p = 1.41 \times 10^{-14}$	12	$p = 3.29 \times 10^{-6}$
11) NA*P*025923-1	6.7	$p = 0.0028$	11	$p = 4.87 \times 10^{-7}$	N/A	$p > 0.05$
12) *Mthfd1l*	6.7	$p = 2.72 \times 10^{-8}$	2.8	$p = 9.65 \times 10^{-5}$	N/A	$p > 0.05$
13) *Elovl3*	6.7	$p = 1.23 \times 10^{-9}$	8.3	$p = 5.32 \times 10^{-15}$	7.1	$p = 6.75 \times 10^{-4}$
14) *C6*	6.3	$p = 1.45 \times 10^{-12}$	10	$p = 1.09 \times 10^{-20}$	9.1	$p = 5.13 \times 10^{-8}$
15) *Meig1*	6.3	$p = 1.50 \times 10^{-5}$	7.1	$p = 4.25 \times 10^{-9}$	7.1	$p = 8.38 \times 10^{-3}$
16) *Tff3*	5.9	$p = 3.27 \times 10^{-6}$	8.3	$p = 2.68 \times 10^{-11}$	N/A	$p > 0.05$
17) *Hsd3b5*	5.6	$p = 9.16 \times 10^{-12}$	9.1	$p = 1.15 \times 10^{-19}$	17	$p = 8.43 \times 10^{-9}$
18) *Dnase1*	5.6	$p = 3.94 \times 10^{-7}$	3.8	$p = 5.20 \times 10^{-7}$	5.6	$p = 3.26 \times 10^{-3}$
19) *Cib3*	5.3	$p = 0.00043$	3.2	$p = 0.0016$	N/A	$p > 0.05$
20) AK046232	5.3	$p = 0.00014$	7.7	$p = 4.62 \times 10^{-9}$	6.7	$p = 9.97 \times 10^{-3}$
21) *Hsd3b4*	5.3	$p = 3.91 \times 10^{-9}$	8.3	$p = 2.44 \times 10^{-16}$	20	$p = 2.95 \times 10^{-8}$
22) *Ntrk1*	5.0	$p = 0.00079$	3.7	$p = 0.00037$	6.3	$p = 0.045$

Abbreviations: B6, C57BL/6J; WT, wild-type; *Esr1*KO, estrogen receptor-α knockout. Fold change represents microarray results from hepatic RNA of mice sacrificed at 9-10 weeks of age. The adjusted *P* value for each transcript is listed. For the *Esr1*KO, the WT females, and the WT males group (B6 background) the microarrays were run with a pool of hepatic RNA. The WT females group (B6 background) is composed of B6 females (n = 18) and the WT female littermates of the estrogen receptor-α (n = 6) and estrogen receptor-β (n = 6) mice. The WT males group (B6 background) is composed of B6 males (n = 15) and WT male littermates of the estrogen receptor-α (n = 6) and estrogen receptor-β (n = 6) mice. *Esr1*KO females are represented by intact individuals (n = 6) and females that underwent a sham operation (n = 6). For the B6;FVB mixed background groups we show results from three individual animals, where the WT males and WT females groups are littermates of the liver estrogen receptor-α knockout (LERKO) animals.

Table 3. Morphometric analysis of mice used in gene expression study.

	No. mice	Body weight (g)	Liver weight (g)	Liver/ body weight	Uterus weight (mg)	Estrogen (pg/ml)
Female wild-type	40	20 ± 1.8 [ab]	0.90 ± 0.091 [b]	0.046 ± 0.0047 [ef]	70 ± 27 [c]	59 ± 76 [ab**]
Ovariectomy + E_2	6	20 ± 0.85 [ab]	1.0 ± 0.078 [cde]	0.051 ± 0.0040 [f]	72 ± 14 [bc]	680 ± 240 [c**]
Ovariectomy	11	23 ± 1.6 [c]	1.0 ± 0.067 [cde]	0.046 ± 0.0035 [def]	28 ± 29 [ab*]	31 ± 9.3 [ab**]
Female *Esr1* heterozygous	6	21 ± 1.3 [bc]	0.85 ± 0.14 [bcd]	0.040 ± 0.0051 [bcd]	82 ± 18 [c]	35 ± 10 [ab]
Female *Esr1* knockout	14	26 ± 2.0 [d]	1.1 ± 0.13 [e]	0.041 ± 0.0045 [ab]	23 ± 8.4 [a]	54 ± 22 [b]
Female LERKO	3	21 ± 1.6 [bc]	1.1 ± 0.18 [bcde]	0.050 ± 0.0049 [cdef]	82 ± 33 [bc]	N/A
Female *Esr2* heterozygous	6	22 ± 0.88 [c]	0.83 ± 0.098 [ab]	0.038 ± 0.0048 [abc]	77 ± 26 [bc]	42 ± 14 [ab]
Female *Esr2* knockout	14	23 ± 1.8 [c]	1.0 ± 0.10 [cde]	0.044 ± 0.0053 [bcde]	82 ± 28 [c]	30 ± 4 [a**]
Male wild-type	27	28 ± 2.5	1.3 ± 0.28	0.047 ± 0.012		
Male *Esr1* knockout	6	29 ± 3.1	1.2 ± 0.27	0.041 ± 0.0079		
Male LERKO	3	27 ± 5.9	1.4 ± 0.48	0.051 ± 0.011		

Abbreviations: E_2, 17β-estradiol; *Esr1*, estrogen receptor-*α*; LERKO, liver estrogen receptor-*α* knockout; *Esr2*, estrogen receptor-β. *Uterine weight was determined for 6 ovariectomized mice. **Serum estrogen levels were measured in 34 of the wild-type females, in 5 of the animals in the ovariectomy plus estrogen group, in 4 of the animals that were ovariectomized, and in 12 of the *Esr2*KO female mice. Body weight, liver weight, liver/body ratio, uterus weight and serum estrogen levels are shown as the mean ± standard deviation. Statistical analysis was carried out separately for male and female groups. Pairwise comparison using Wilcoxon rank sum test (2-sided) with correction for multiple samples (FDR) was used to test for differences and a $P < 0.05$ was considered significant. Different letters indicate statistically significant differences between groups. The female wild-type group is composed of intact C57BL/6J (B6) females, B6 females that underwent a sham operation (with or without subcutaneous placebo), as well as wild-type littermates of *Esr1*, *Esr2*, and LERKO mice. Serum was not collected from the LERKO females or their wild-type littermates. The *Esr1* and *Esr2* knockout female groups consist of both intact and mice that underwent a sham operation. The wild-type male group is composed of B6 males as well as wild-type littermates of *Esr1*, *Esr2*, and the LERKO mice.

Table S4. Transcripts expressed differentially in C57BL/6J females between the ovariectomized group vs. the ovariectomy plus 17β-estradiol add-back group and corresponding sex-specific and estrogen receptor-*α* dependent expression.

	Ovariectomy vs. Ovariectomy plus 17β-estradiol		Wild-type females vs. Wild-type males		Wild-type females vs. *Esr1*KO females	
**Higher in ovariectomy plus 17β-estradiol**	**Fold Change**	**Adjusted p-value**	**Fold Change**	**Adjusted p-value**	**Fold Change**	**Adjusted p-value**
Tff3	34	$p = 1.29×10^{-5}$	Male > Female 8.6	$p = 2.68×10^{-11}$	*Esr1*KO > WT 6.0	$p = 3.27×10^{-6}$
2810439F02Rik	31	$p = 9.82×10^{-5}$	Male > Female 5.1	$p = 0.0022$	*Esr1*KO > WT 3.8	$p = 0.0022$
Osbpl3	7.7	$p = 0.027$	Male > Female 2.8	$p = 0.00021$	*Esr1*KO > WT 3.8	$p = 0.00020$
Akr1c18	6.4	$p = 0.049$	Female > Male 4.3	$p = 1.18×10^{-7}$	WT > *Esr1*KO 3.1	$p = 0.0016$
Epdr1	5.9	$p = 0.00025$	Female > Male 2.4	$p = 7.67×10^{-7}$	N/A	$p > 0.05$
EG627821	4.8	$p = 0.0078$	Male > Female 2.2	$p = 2.42×10^{-5}$	N/A	$p > 0.05$
Cyp7b1	4.5	$p = 0.0073$	Male > Female 6.3	$p = 4.27×10^{-17}$	*Esr1*KO > WT 2.9	$p = 5.08×10^{-6}$
Bmper	4.1	$p = 0.0013$	Female > Male 3.8	$p = 8.89×10^{-15}$	N/A	$p > 0.05$

Higher in ovariectomy	Fold Change	Adjusted *p*-value	Fold Change	Adjusted *p*-value	Fold Change	Adjusted *p*-value
Hspa1a	6.3	*p* = 0.046	Male > Female 2.7	*p* = 0.0013	*Esr1*KO > WT 3.6	*p* = 0.0017

Abbreviations: *Esr1*KO, estrogen receptor-α knockout; WT, wild-type. Transcripts that differed between the ovariectomy group and the group that received ovariectomy plus 17β-estradiol add-back, that were also either estrogen receptor-α dependent or sex-specific. Fold change represents microarray results from hepatic RNA pools from individual mice sacrificed at 9-10 weeks of age. The 17β-estradiol group is composed of ovariectomized C57BL/6J (B6) mice with 17β-estradiol (0.1 mg pellet) add-back (n = 6). The ovariectomized group is composed of B6 mice that underwent ovariectomy alone (n = 5) or ovariectomy plus placebo (n = 6). The female wild-type (WT) group is composed of intact B6 females (n = 18) and the WT female littermates of the estrogen receptor-α (n = 6) and estrogen receptor-β mice (n = 6). WT males are represented by B6 males (n = 15) and WT littermates of estrogen receptor-α (n = 6) and estrogen receptor-β mice (n = 6). *Esr1*KO females are represented by intact individuals (n = 6) and females that underwent a sham operation (n = 6).

Table S5. Ovarian hormon×10−de*p*endent transcri*p*ts in C57BL/6J mice which are also de*p*endent on 17β-estradiol.

	**Ovariectomy > Intact females**		**Ovariectomy > Ovariectomy *p*lus 17β-estradiol**	
	**Fold Change**	**Adjusted *p*-value**	**Fold Change**	**Adjusted *p*-value**
1) *Ascc3l1*	13	$p = 0.00027$	17	$p = 0.0095$
2) AK048398	12	$p = 8.62\times10^{-6}$	12	$p = 0.0029$
3) *Ppp1r1a*	8.7	$p = 0.00016$	8.4	$p = 0.027$
4) *Lphn3*	6.9	$p = 0.00014$	6.7	$p = 0.023$
5) *Myb*	5.6	$p = 0.00081$	6.0	$p = 0.042$
6) AK033751	5.6	$p = 0.00014$	5.6	$p = 0.018$
7) *Nipbl*	5.6	$p = 1.27\times10^{-8}$	5.6	$p = 4.09\times10^{-5}$
8) *Nefl*	4.9	$p = 0.00014$	5.0	$p = 0.018$
9) AK036362	4.6	$p = 3.11\times10^{-9}$	4.7	$p = 1.20\times10^{-5}$
10) 4930522N08Rik	3.4	$p = 9.94\times10^{-5}$	3.3	$p = 0.018$
11) D930020E02Rik	2.4	$p = 1.93\times10^{-5}$	2.4	$p = 0.0045$
12) *Edil3*	2.3	$p = 4.15\times10^{-5}$	2.4	$p = 0.0063$
13) A730076H11Rik	2.3	$p = 0.00091$	2.5	$p = 0.027$

	**Intact females > Ovariectomy**		**Ovariectomy *p*lus 17β-estradiol > Ovariectomy**	
	**Fold Change**	**Adjusted *p*-value**	**Fold Change**	**Adjusted *p*-value**
1) 2610018I03Rik	2.4	$p = 3.71\times10^{-5}$	2.8	$p = 0.0010$
2) *Ard1*	2.3	$p = 0.00019$	2.3	$p = 0.019$
3) *Nmd3*	2.3	$p = 0.00014$	2.2	$p = 0.011$
4) *Vps13b*	2.1	$p = 3.94\times10^{-7}$	2.1	$p = 0.00029$

Transcri*p*ts that are altered with ovariectomy and also de*p*endent on17β-estradiol add-back. Fold change re*p*resents microarray results from he*p*atic RNA *p*ools from individual C57BL/6J (B6) mice sacrificed at 9-10 weeks of age. The female intact grou*p* is com*p*osed of B6 females (n = 18), and the wild-ty*p*e littermates of the estrogen rece*p*tor-α (n = 6) and estrogen rece*p*tor-β (n = 6) mice. The ovariectomized grou*p* is com*p*osed of B6 mice that underwent ovariectomy alone (n = 5) or ovariectomy *p*lus a subcutaneous *p*lacebo *p*ellet (n = 6). The ovariectomy *p*lus 17β-estradiol grou*p* is com*p*osed of ovariectomized mice with subcutaneous estrogen (0.1 mg *p*ellet) add-back (n = 6).

Table S6. Transcri*p*ts with at least 2-fold differential ex*p*ression between LERKO females and wild-ty*p*e females and corres*p*onding sex-s*p*ecific ex*p*ression.

	**(B6;FVB background) WT females vs. LERKO females**		**(B6;FVB background) WT females vs. WT males**		**(B6 background) WT females vs. WT males**	
**Higher in WT (B6;FVB) females**	**Fold Change**	**Adjusted *p*-value**	**Fold Change**	**Adjusted *p*-value**	**Fold Change**	**Adjusted *p*-value**
1) *Ifi202b*	27	$p = 0.0038$	Fem > Male: 10×	$p = 0.025$	N/A	$p > 0.05$
2) 3110004L20Rik	8.7	$p = 0.0034$	Fem > Male: 7.9 ×	$p = 0.011$	N/A	$p > 0.05$
3) 4930573O21Rik	6.2	$p = 0.0037$	N/A	$p > 0.05$	Male > Fem: 1.9 ×	$p = 0.016$
4) *Esr1*	6.0	$p = 0.00015$	Fem > Male: 3.1 ×	$p = 0.014$	Fem > Male: 4.4 ×	$p = 2.78\times10^{-16}$
5) LOC14210	5.9	$p = 0.0025$	N/A	$p > 0.05$	N/A	$p > 0.05$
6) *Gabra3*	5.8	$p = 0.0014$	Fem > Male: 4.6 ×	$p = 0.0013$	N/A	$p > 0.05$

7) *Slit2*	5.7	$p = 0.00061$	N/A	$p > 0.05$	N/A	$p > 0.05$
8) *Tnfrsf9*	5.1	$p = 0.034$	N/A	$p > 0.05$	N/A	$p > 0.05$
9) AK033775	4.7	$p = 0.033$	N/A	$p > 0.05$	N/A	$p > 0.05$
10) *Dbc1*	4.7	$p = 0.0057$	Fem > Male: 2.8 ×	$p = 0.040$	N/A	$p > 0.05$
11) AK086393	4.6	$p = 0.028$	N/A	$p > 0.05$	N/A	$p > 0.05$
12) *Vdr*	4.2	$p = 0.019$	N/A	$p > 0.05$	N/A	$p > 0.05$
13) *Ccr3*	3.7	$p = 0.038$	N/A	$p > 0.05$	N/A	$p > 0.05$
14) *Txlnb*	3.4	$p = 0.034$	N/A	$p > 0.05$	N/A	$p > 0.05$
15) 4933426K21Rik	3.4	$p = 0.0078$	N/A	$p > 0.05$	N/A	$p > 0.05$
16) NAP027952-1	3.2	$p = 0.0062$	N/A	$p > 0.05$	N/A	$p > 0.05$
17) *Pcdh19*	3.2	$p = 0.033$	N/A	$p > 0.05$	N/A	$p > 0.05$
18) *Ptgfr*	3.2	$p = 0.0019$	Fem > Male: 2.8 ×	$p = 0.0013$	N/A	$p > 0.05$
19) *Olfr1294*	3.0	$p = 0.040$	N/A	$p > 0.05$	N/A	$p > 0.05$
20) *Ptprr*	3.0	$p = 0.015$	N/A	$p > 0.05$	N/A	$p > 0.05$
21) 1500015O10Rik	2.9	$p = 0.0079$	N/A	$p > 0.05$	N/A	$p > 0.05$
22) *Pdzrn3*	2.9	$p = 0.015$	N/A	$p > 0.05$	Fem > Male: 1.5 ×	$p = 0.018$
23) 1110028C15Rik	2.8	$p = 0.00071$	N/A	$p > 0.05$	N/A	$p > 0.05$
24) AK019816	2.8	$p = 0.020$	N/A	$p > 0.05$	N/A	$p > 0.05$
25) AK029741	2.7	$p = 0.0084$	N/A	$p > 0.05$	N/A	$p > 0.05$
26) *Dpep2*	2.7	$p = 0.0088$	N/A	$p > 0.05$	N/A	$p > 0.05$
27) *Ccdc68*	2.7	$p = 0.016$	Fem > Male: 6.1 ×	$p = 9.36\times10^{-9}$	Fem > Male: 1.8 ×	$p = 0.00017$
28) A930037H05Rik	2.6	$p = 0.049$	N/A	$p > 0.05$	N/A	$p > 0.05$
29) *Calb1*	2.6	$p = 0.030$	N/A	$p > 0.05$	Male > Fem: 1.6 ×	$p = 0.045$
30) *Hsd11b2*	2.5	$p = 0.015$	N/A	$p > 0.05$	N/A	$p > 0.05$
31) *Cd34*	2.4	$p = 0.028$	Fem > Male: 2.1 ×	$p = 0.024$	Fem > Male: 1.8 ×	$p = 5.99\times10^{-5}$
32) *Cd200r3*	2.4	$p = 0.019$	N/A	$p > 0.05$	N/A	$p > 0.05$
33) *Tub*	2.4	$p = 0.00012$	N/A	$p > 0.05$	N/A	$p > 0.05$
34) *Kcne1*	2.3	$p = 0.011$	N/A	$p > 0.05$	N/A	$p > 0.05$
35) *Lbxcor1*	2.3	$p = 0.028$	N/A	$p > 0.05$	N/A	$p > 0.05$
36) *Rasa1*	2.3	$p = 0.016$	N/A	$p > 0.05$	N/A	$p > 0.05$
37) *Tigd4*	2.2	$p = 0.034$	N/A	$p > 0.05$	N/A	$p > 0.05$
38) *Chgb*	2.2	$p = 0.0038$	N/A	$p > 0.05$	N/A	$p > 0.05$
39) AF087578	2.1	$p = 0.016$	Fem > Male: 1.8 ×	$p = 0.018$	N/A	$p > 0.05$
40) *Prl5a1*	2.1	$p = 0.011$	N/A	$p > 0.05$	N/A	$p > 0.05$
41) *Gpm6a*	2.0	$p = 0.023$	N/A	$p > 0.05$	N/A	$p > 0.05$

	**(B6;FVB background) WT females vs. LERKO females**		**(B6;FVB background) WT females vs. WT males**		**(B6 background) WT females vs. WT males**	
**Higher in LERKO females**	**Fold Change**	**Adjusted *p*-value**	**Fold Change**	**Adjusted *p*-value**	**Fold Change**	**Adjusted *p*-value**
1) *Ms4a4b*	15	$p = 0.0090$	Fem > Male: 1.7 ×	$p = 0.017$	Fem > Male: 1.6 ×	$p = 0.0030$
2) *Stx3*	7.1	$p = 0.00020$	Male > Fem: 2.9x	$p = 0.044$	N/A	$p > 0.05$
3) AK086941	6.5	$p = 1.39\times10^{-6}$	Male > Fem: 2.4 ×	$p = 0.035$	N/A	$p > 0.05$
4) D230034L24Rik	4.6	$p = 0.0062$	N/A	$p > 0.05$	N/A	$p > 0.05$
5) *Hbb-b1*	4.3	$p = 0.023$	N/A	$p > 0.05$	N/A	$p > 0.05$
6) *Tanc2*	4.2	$p = 0.036$	Male > Fem: 3.3 ×	$p = 0.035$	N/A	$p > 0.05$
7) *Gja5*	4.1	$p = 0.019$	Male > Fem: 5.8 ×	$p = 0.00011$	N/A	$p > 0.05$
8) 4921511K06Rik	3.8	$p = 0.0067$	N/A	$p > 0.05$	N/A	$p > 0.05$
9) *Styxl1*	3.6	$p = 0.028$	Male > Fem: 2.8 ×	$p = 0.033$	N/A	$p > 0.05$
10) AK041868	3.5	$p = 0.033$	N/A	$p > 0.05$	N/A	$p > 0.05$

11) 6330549D23Rik	3.3	$p = 0.034$	Male > Fem: 4.4 ×	$p = 0.00034$	N/A	$p > 0.05$
12) *Rims4*	3.3	$p = 0.025$	N/A	$p > 0.05$	N/A	$p > 0.05$
13) ENSMUSG00000060915	3.3	$p = 0.0038$	N/A	$p > 0.05$	N/A	$p > 0.05$
14) *Gjb4*	3.2	$p = 0.028$	N/A	$p > 0.05$	N/A	$p > 0.05$
15) *Ankrd2*	3.1	$p = 0.012$	N/A	$p = 0.0078$	N/A	$p > 0.05$
16) 1700016B15Rik	2.9	$p = 0.018$	N/A	$p > 0.05$	N/A	$p > 0.05$
17) AK029708	2.8	$p = 0.020$	N/A	$p > 0.05$	N/A	$p > 0.05$
18) AK037078	2.8	$p = 0.016$	N/A	$p > 0.05$	N/A	$p > 0.05$
19) 9430022F06Rik	2.7	$p = 0.028$	N/A	$p > 0.05$	N/A	$p > 0.05$
20) AK088873	2.7	$p = 0.0021$	N/A	$p > 0.05$	N/A	$p > 0.05$
21) *Pvr*	2.7	$p = 0.023$	Fem > Male: 2.4 ×	$p = 6.25{\times}10^{-8}$	Fem > Male: 1.9 ×	$p = 6.52{\times}10^{-15}$
22) *Pax1*	2.5	$p = 0.028$	Fem > Male: 3.5 ×	$p = 0.035$	N/A	$p > 0.05$
23) *Npc1*	2.4	$p = 2.31{\times}10^{-7}$	N/A	$p > 0.05$	N/A	$p > 0.05$
24) AK040918	2.4	$p = 0.016$	N/A	$p > 0.05$	N/A	$p > 0.05$
25) 6430562O15Rik	2.3	$p = 0.028$	N/A	$p > 0.05$	N/A	$p > 0.05$
26) *Pan3*	2.2	$p = 0.015$	N/A	$p > 0.05$	N/A	$p > 0.05$
27) AK039103	2.1	$p = 0.0026$	N/A	$p > 0.05$	N/A	$p > 0.05$
28) *Ctsf*	2.1	$p = 0.018$	N/A	$p > 0.05$	N/A	$p > 0.05$
29) 5730507N06Rik	2.0	$p = 0.023$	Male > Fem: 1.9 ×	$p = 0.015$	N/A	$p > 0.05$

Abbreviations: B6, C57BL/6J; WT, wild-type; LERKO, liver estrogen receptor-α knockout. Fold change represents microarray results from hepatic RNA of mice sacrificed at 9-10 weeks of age. The adjusted *p*-value for each transcript is listed. For the LERKO females vs. WT females (B6;FVB) comparison, and the WT sex-specific (B6;FVB background) comparison, these results are from three individual animals from each group. The B6;FVB WT animals were littermates of the LERKO animals. The WT sex-specific (B6 background) comparison represent pools of hepatic RNA. The female B6 WT group is composed of B6 females (n = 18) and the WT female littermates of the global estrogen receptor-α (n = 6) and estrogen receptor-β mice (n = 6). The B6 WT males group is composed of B6 males (n = 15) and the WT male littermates of the global estrogen receptor-α (n = 6) and estrogen receptor-β (n = 6) mice.

Table S7. Quantitative real-time PCR primers used to measure hepatic mRNA levels in global estrogen receptor-α knockout females and wild-type littermates.

Gene Symbol	Primer Sequence (5'-3')	Accession Number	Efficiency	Amplicon (bp)
Actb	F) 5′-CCCTGAGGGTCTTTTCCAG-3<br>R) 5′-GGATGCCACAGGATTCCATA-3′	NM_007393.1	2.0	53
Fmo3	F) 5′-AACTATGGTTTGGTGCCTTT-3′<br>R) 5′-ATTGAACACGGGCTCTTT-3′	NM_008030.1	2.1	54

Sult3a1	F) 5′-TGCAAACATTTCTA-GATGGAG-3′ R) 5′-TACCAGCCTCTTATGTGGTC-3′	NM_020565.1	2.1	64
3β-Hsd4/5	F) 5′-TCCAGACAGAC-CATCCTAGA-3′ R) 5′-ACACAAGCATCCAGTAG-GAG-3′	NM_001111336.1/ NM_008295.1	1.8	62
Cyp4a12	F) 5'-CTCATTCCTGCCCTTCTC-3' R) 5'-AGCTCATTCATCGCAAACT-3'	NM_172306.1	2.2	66

Abbreviations: *Actb*, β-actin; *Fmo3*, flavin-containing monooxygenase 3; *Sult3a1*, sulfotransferase 3a1; *3β-Hsd4/5*, 3β-hydroxysteroid dehydrogenase 4/5; *Cyp4a12*, cytochrome *p*450 4a12.
